# Phenotypic Heterogeneity and the Evolution of Bacterial Life Cycles

**DOI:** 10.1371/journal.pcbi.1004764

**Published:** 2016-02-19

**Authors:** Jordi van Gestel, Martin A. Nowak

**Affiliations:** 1 Program for Evolutionary Dynamics, Departments of Organismic and Evolutionary Biology and Mathematics, Harvard University, Boston, Massachusetts, United States of America; 2 Theoretical Research in Evolutionary Life Sciences, Groningen Institute for Evolutionary Life Sciences, University of Groningen, Groningen, the Netherlands; University of Texas at Austin, UNITED STATES

## Abstract

Most bacteria live in colonies, where they often express different cell types. The ecological significance of these cell types and their evolutionary origin are often unknown. Here, we study the evolution of cell differentiation in the context of surface colonization. We particularly focus on the evolution of a ‘sticky’ cell type that is required for surface attachment, but is costly to express. The sticky cells not only facilitate their own attachment, but also that of non-sticky cells. Using individual-based simulations, we show that surface colonization rapidly evolves and in most cases leads to phenotypic heterogeneity, in which sticky and non-sticky cells occur side by side on the surface. In the presence of regulation, cell differentiation leads to a remarkable set of bacterial life cycles, in which cells alternate between living in the liquid and living on the surface. The dominant life stage is formed by the surface-attached colony that shows many complex features: colonies reproduce via fission and by producing migratory propagules; cells inside the colony divide labour; and colonies can produce filaments to facilitate expansion. Overall, our model illustrates how the evolution of an adhesive cell type goes hand in hand with the evolution of complex bacterial life cycles.

## Author Summary

In nature, most bacteria occur in surface-attached colonies. Inside these colonies, cells often express many different phenotypes. The significance of these phenotypes often remains unknown. We study the evolution of cell differentiation in the context of surface colonization. We particularly focus on the evolution of a ‘sticky’ cell type that is needed for surface attachment. We show that the sticky cell type readily evolves and escapes from competition in the liquid by attaching to the surface. In most cases, surface colonization is accompanied by phenotypic heterogeneity, in which sticky and non-sticky cell co-occupy the surface. The non-sticky cells hitchhike with the sticky cells, thereby profiting from surface attachment without paying the cost of being sticky. In the presence of regulation, cell differentiation leads to the evolution of intricate bacterial life cycles in which cells alternate between living in surface-attached colonies and living in the liquid. The bacterial life cycles are orchestrated by temporal and spatial pattern formation of cell types. Our model illustrates how cell differentiation can be of key importance for the evolution of bacterial life cycles.

## Introduction

In nature, most bacteria live in surface-attached colonies [1,2]. Inside these colonies, cells typically express a remarkable diversity of phenotypes [3,4]. This phenotypic heterogeneity can be induced by genetic mutations, inherent stochasticity or the environment [3–7]. For example, during colony growth in *Pseudomonas aeruginosa*, genetic changes result in phenotypic heterogeneity [8]. Some cells express accelerated colony development, others have higher abilities to disseminate and yet others increase the resistance of the colony to environmental stressors. In *Pseudomonas putida* inherent stochasticity in the expression of a quorum-sensing signal leads to phenotypic heterogeneity. Some cells express the quorum-sensing signal and consequently disperse away from the colony, while others do not and remain tightly attached [9]. Probabilistic cell differentiation also influences the onset of colony formation. In *Bacillus subtilis*, motile cells can stochastically differentiate into matrix-producing cell chains, which can adhere to the surface [10–13]. Even inside a *B*. *subtilis* colony, matrix production can be heterogeneously expressed, in which only a fraction of cells expresses matrix [11,13–17]. Since matrix can be shared between cells, it is often hypothesized that cells divide labour [15,18,19]: some cells produce matrix, while others specialize on complementary tasks (for an example of heterogeneous matrix expression in *B*. *subtilis* see S1 Text and S1 Fig).

Adhesive cells, like the matrix-producing cells in *B*. *subtilis*, are critical to surface colonization, because they allow for cell-to-surface and cell-to-cell adhesion [20–23]. In the lab, surface colonization readily evolves *de novo*. For example, when *Pseudomonas fluorescens* is grown in static liquid culture, cells evolve matrix production in order to colonize the air-liquid interface [24–26], where oxygen is available for aerobic respiration. The adhesive molecules that allow for colony formation can also trap cells inside the colony and, hence, prevent them from dispersing. Nadell and Bassler [27] demonstrated this in *Vibrio cholera* by growing matrix-producing and matrix-deficient cells together in a flow chamber. Whereas matrix-producing cells are more effective in colonizing the surface than matrix-deficient cells, they are strongly outnumbered by the latter in terms of propagule production. The same trade-off between surface colonization and dispersal was also apparent in an experiment of Poltak and colleagues [28,29]. They evolved *Burkholderia cenocepacia* cells for consecutive rounds of surface colonization and dispersal. Cells were grown in test tubes, were they could colonize a submerged plastic bead. Every day, the bead was transferred to a new test tube that contained a yet un-colonized bead, which was the next to be transferred. Thus, every day, cells had to disperse from their original bead and colonize the new one. Over evolutionary time, colony variants evolved that differed in their capacity to colonize and disperse: the variants that could easily colonize the surface were bad in dispersing and *vice versa*. In a recent experiment of Hammerschmidt and colleagues [30], a population of *Pseudomonas fluorescens* was forced to go through consecutive rounds of surface attachment at the air-liquid interface and surface detachment. This resulted in the evolution of a genetic switch that through slipped-strand mispairing produced alternating phenotypes that could colonize the surface by over-producing an adhesive molecule or detach to the liquid. In other words, evolution resulted in a bacterial life cycle in which cells alternated between living on the surface and living in the liquid. Altogether, the above experiments show that adhesive cell types control surface colonization and dispersal.

Even though numerous models have examined how bacterial cells, including matrix-producing cells, can interact on a surface [19,31–39], few models have examined the evolution of cells in a dynamical environment where cells can alternative between living on a surface and living in the liquid [40]. In this study, we examine the evolution of adhesive cells in the context of surface colonization. Inspired by the above experiments, we constructed an individual-based model in which cells can evolve a ‘sticky’ phenotype. The model does not enforce a particular life cycle, but instead cells can ‘choose’ to become sticky and adhere to the surface or remain non-sticky and stay in the liquid. Sticky cells not only facilitate their own attachment, but also that of other cells. We implement three model variants to examine alternative induction mechanisms of cell differentiation. For all model variants, surface colonization readily evolves, which in most cases is accompanied by phenotypic heterogeneity, where sticky and non-sticky cells occur side by side on the surface. In the presence of regulation, phenotypic heterogeneity orchestrates the bacterium´s life cycle by affecting a colony´s survival rate, expansion rate and propagule production. Our model therefore illustrates that the evolution of cell differentiation goes hand in hand with that of a bacterium’s life cycle.

### Model structure

As illustrated by the examples above, cells can colonize a wide range of surfaces: including the air-liquid interface [26,41,42], air-solid interface and liquid-solid interface–e.g. plant roots [43–46], soil particles [47,48], fungi [49]. At these surfaces, attachment is governed by distinct biophysical mechanisms, although generally speaking adhesive cells are acquired. For simplicity, our model ignores the biophysical details of attachment and simply assumes that adhesive cells can adhere to the surface, whereas non-adhesive cells cannot. As such, the model does not resemble any specific type of surface colonization, instead we aim to make a first step in exploring how adhesive cell types evolve in a dynamical environment where cells can attach and detach from a surface at any moment in time.

**Fig 1:**
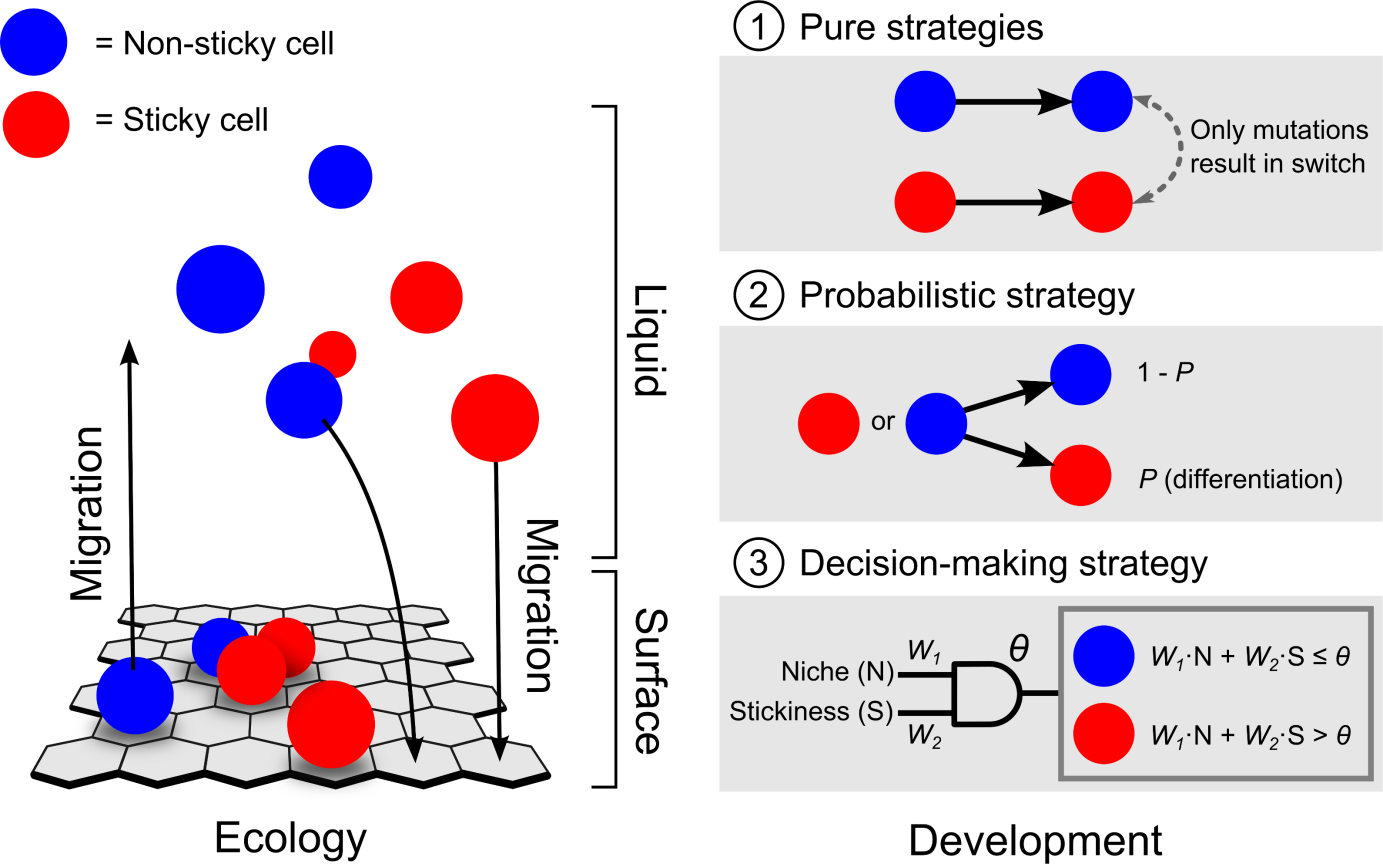
Model structure. The model structure consists of two parts: ecology and development. Ecology: cells can express two phenotypes, non-sticky cells or sticky cells. The sticky and non-sticky cells occur in one of two niches: the liquid or the surface. Cells in liquid are well mixed. Cells on the surface are placed on hexagonal grid and can only stay on the surface when being sticky or directly surrounded by sticky cell. Cells can migrate from the liquid to the surface and *vice versa*. Migration to the surface is only possible when being sticky or when a cell is directly surrounded by sticky cell on the surface. Development: three differentiation strategies are examined in the model: (1) pure strategy, (2) probabilistic strategy, (3) decision-making strategy. In the pure strategy, cells only express one phenotype and can switch via mutations. In the probabilistic strategy, cells have a probability *P* to differentiate. In the decision-making strategy, cells can differentiate in response to the environment. Cells sense two environmental cues: the niche in which they occur (N) and the fraction of surrounding sticky cells (i.e. stickiness, S). Cells differentiate when the sum of regulatory input, weighted by connection weights (*W*_*1*_ and *W*_*2*_), exceeds the activation threshold (θ).

We assume that the model consists of two environments: the liquid and the surface (Fig 1). At the onset of evolution, cells only occur in the liquid. Cells can express two cell types: sticky and non-sticky cells. Sticky cells have a reduced cell division rate (*R*) and are required for surface attachment. They can attach to any unoccupied position on the surface. Non-sticky cells can also attach to the surface, but only when immediately neighbouring a sticky cell. In other words, non-sticky cells can hitchhike with sticky cells, like observed in the lab (e.g. S1 Text, S1C and S1D Fig). The surface consists of a two-dimensional hexagonal grid, so each sticky cell can have at most six non-sticky neighbours (Fig 1). Surface attachment is beneficial, because it allows cells to escape from competition in the liquid. This benefit is present as long as there is space available on the surface. At the same time, surface attachment requires sticky cells that carry the cost of a lower cell division rate. Non-sticky cells that attach to the surface by hitchhiking with sticky cells escape from the costs of being sticky, but still have the benefits of surface attachment.

We examine the evolution of sticky cells for three model variants. These variants differ with respect to the differentiation strategy that evolves (Fig 1): cells either have a (1) pure strategy, (2) probabilistic strategy, (3) decision-making strategy. In the pure strategy, cells can only switch between being sticky and non-sticky by mutations. As a result, each genotype expresses one phenotype. In the probabilistic strategy, cells differentiate with a certain probability (*P*). This probability can change over evolutionary time, by the accumulation of mutations (for details see Material and Methods). In the decision-making strategy, cells can differentiate in response to the environment. Cells sense two environmental cues: the niche in which they occur (N = 0 in the liquid and N = 1 on the surface) and the fraction of sticky cells (i.e. stickiness, S). On the surface, cells only sense the fraction of sticky cells in the neighbouring positions on the grid. In the liquid, the fraction of sticky cells is determined with respect to the entire population. The sensory input to a cell is weighted by so-called connection weights (*W*_*1*_ and *W*_*2*_). When the sum of regulatory input exceeds a given threshold (θ) a cell differentiates to a sticky cell. Over evolutionary time the connection weights and activation threshold can evolve (see Fig 1 and Material and Methods).

For each model variant, we start evolution with a population of non-sticky cells in the liquid. All genotypic variables are set to zero (model variant 2: *P* = 0, and model variant 3: *W*_*1*_ = *W*_*2*_ = θ = 0). Cells can evolve for 400.000 time step. At each time step, one of the following events can occur (see Material and Methods): (i) migration to the surface, (ii) migration to the liquid, (iii) cell differentiation, (iv) cell death, (v) cell division. The event that occurs is chosen randomly. We explore the outcome of evolution by varying two modelling parameters: *R* and *P*_*m*_. *R* is the relative cell division rate of sticky cells. When the costs of being sticky are high, sticky cells cannot divide (*R* = 0) and, when the costs are low, sticky and non-sticky cells are equally likely to divide (*R* = 1). *P*_*m*_ is the probability to migrate to the surface. As default setting *P*_*m*_ = 0.1, which means that cells have a 10% probability to migrate to a random location on the surface. This does not mean that they necessarily attach to this particular location. A cell can only attach when the randomly chosen position on the surface is vacant and the cell is sticky or surrounded by a sticky cell.

## Results

### Surface colonization of three differentiation strategies

**Fig 2:**
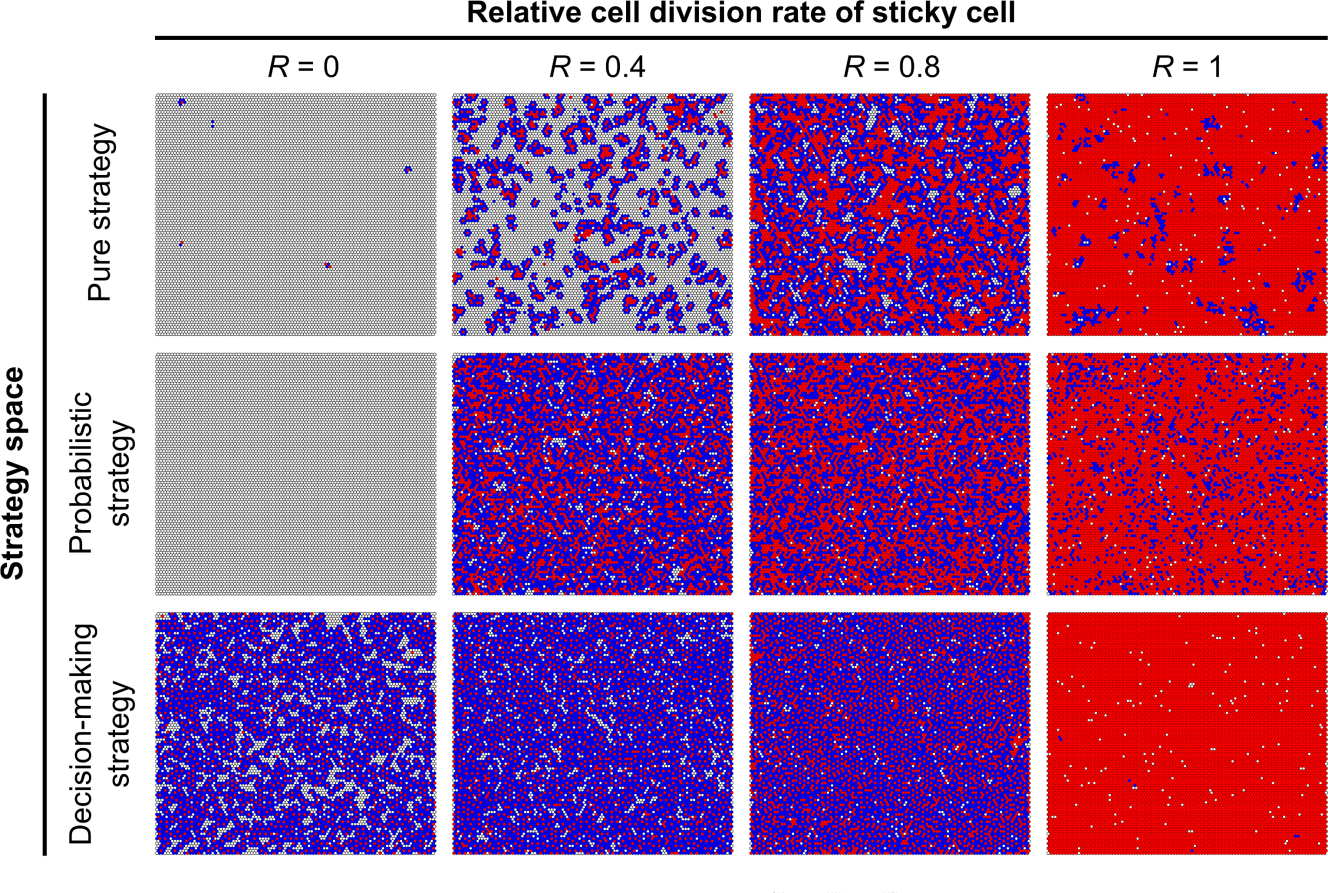
Colonization of surface at end of evolution. Representative surfaces at the end of evolution with sticky cells (red) and non-sticky cells (blue). Cells are attached to a hexagonal grid (black lines). Surfaces are shown for independent simulations at different relative cell division rates of sticky cells (*R*) and for the three differentiation strategies (see Fig 1).

**Fig 3:**
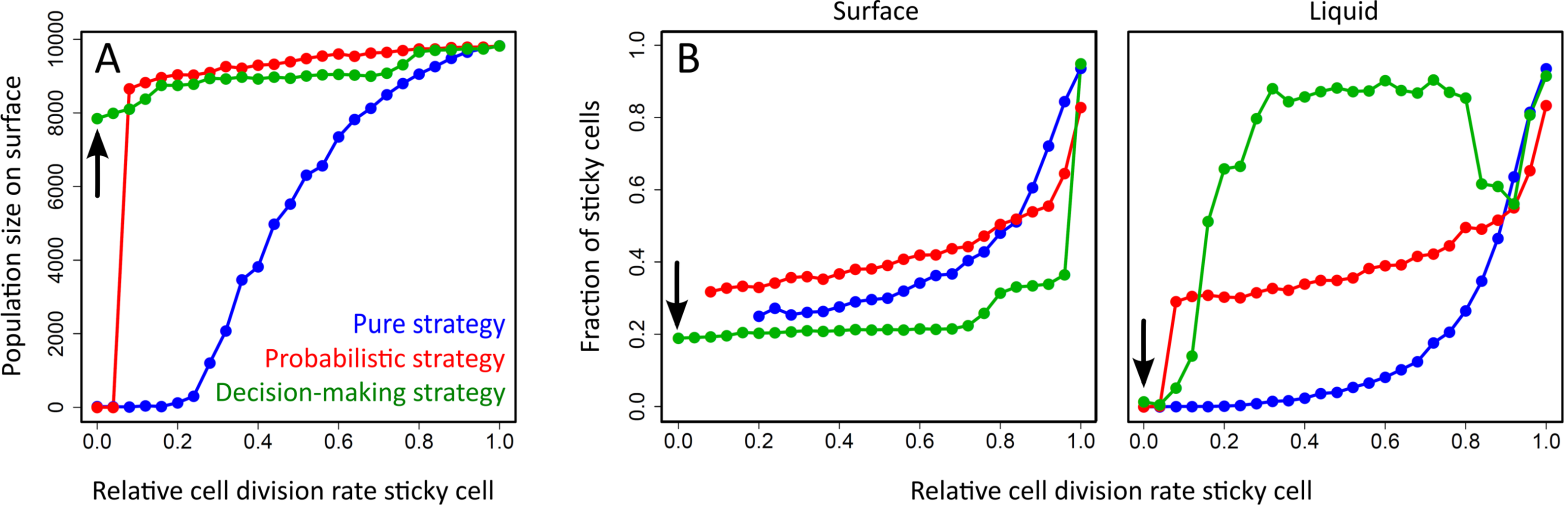
Population size and fraction of sticky cells on surface and in liquid. (A) Population size on surface for three differentiation strategies (blue = pure strategy, red = probabilistic strategy, green = decision-making strategy) at different relative cell division rates of sticky cells (*R*). The carrying capacity of surface is 10.000 cells. (B) Fraction of sticky cells on surface and in liquid. Fraction of sticky cells on surface is only calculated for the simulations in which more than 100 cells attached to the surface. For low cell division rates of the sticky cell, only the decision-making cells attached to the surface. The black arrows in (A) and (B) point to simulations in which the sticky cells could not divide (*R* = 0). This condition is further examined in Fig 4 and Fig 5.

We first examined the evolution of surface colonization at various relative growth rates of the sticky cells (*R* = 0, 0.4, 0.8, 1). Fig 2 shows some representative surfaces at the end of evolution, for the pure, probabilistic and decision-making strategy. Sticky cells are shown in red and the non-sticky cells in blue. The three differentiation strategies differ in their capacity to colonize the surface (see also Fig 3A). The pure strategy shows some surface colonization at all cost levels, but the number of cells on the surface is very low at high costs of being sticky, i.e. low cell division rates of sticky cells (*R*). The few sticky cells that occupy the surface at *R* = 0 express a maladaptive phenotype, because these cells cannot reproduce, nor can they switch phenotype (in the pure strategy, cells can only switch phenotype through mutations that occur during cell division). The probabilistic strategy evolved a nearly full surface colonization at most costs, but cannot colonize the surface at the highest costs (*R* = 0). The decision-making strategy can colonize the surface at all costs; even when sticky cells cannot divide (*R* = 0).

The fraction of sticky cells on the surface decreases for higher costs of being sticky. Moreover, the fraction of sticky cells is lower in colonies from the decision-making strategy than in colonies from either the probabilistic or pure strategy. In the decision-making strategy, cells furthermore show spatial pattern formation (Fig 2). At *R* = 0 and *R* = 0.4, sticky cells only have non-sticky neighbours. In other words, the sticky cells–together with their non-sticky neighbours–form separated islands on the surface. At *R* = 0.8, sticky cells also form filaments. Filaments are short concatenations of sticky cells (2–8 sticky cells), which are surrounded by non-sticky neighbours. Only in a few cases do sticky cells also clump together. When there are no costs of being sticky (*R* = 1), all cells express the sticky phenotype and there is no spatial pattern formation. There are no regular spatial patterns for the probabilistic and pure strategy, because these strategies cannot account for the number of sticky neighbours.

### The fraction of sticky cells on the surface and in the liquid

Next, we examined the fraction of sticky cells on the surface and in the liquid (Fig 3B). On the surface, as shown by Fig 2, the decision-making strategy produces the lowest fraction of sticky cells. In the pure and probabilistic strategies, the fraction of sticky cells gradually increases with higher *R* values. In other words, at lower costs of being sticky, a larger fraction of cells expresses the sticky phenotype. In the decision making strategy, the fraction of sticky cells does not change gradually with *R*. Instead, the fraction of sticky cells is around 20% (*R* = 0–0.72), 30% (*R* = 0.8–0.96) or 100% (*R* = 1). These levels correspond to distinct spatial patterns observed in Fig 2: the 20% sticky cells correspond to isolated islands of sticky cells; the 30% sticky cells correspond to short filaments; and the 100% sticky cells correspond to clumps of sticky cells.

The fraction of sticky cells in the liquid differs from that on the surface (Fig 3B). For the pure strategy the difference is small. The fraction of sticky cells in the liquid is lower than that on the surface, due to the high cell division rate of non-sticky cells. Sticky cells that migrate to the surface remain attached until they die. Despite this permanent attachment, there is still a relatively large fraction of sticky cells in the liquid (especially for high *R* values), because surface-attached sticky cells can dislodge their daughter cells to the liquid after cell division (we assume that the colony is flat, so any cell division in the z-direction would result in the migration of a cell to the liquid; see Material and Methods). For the probabilistic strategy, the fractions of sticky cells in the liquid and on the surface are nearly the same. Even though there is a selective advantage for non-sticky cells in the liquid (i.e. higher cell division rate), this does not affect the frequency of sticky cells too much, because all cells have a given probability to become sticky. For the decision-making strategy, we observed a surprisingly large difference between the fraction of sticky cells in the liquid and on the surface. At very low and very high costs of sticky cells (*R* ≈ 1 or *R* ≈ 0), the fraction of sticky cells in the decision-making strategy is more or less the same as that for the pure and probabilistic strategies, but at intermediate costs (*R* = 0.2–0.8) almost 90% of the cells in the liquid are sticky.

How can there be so many sticky cells in the liquid, while these cells have a lower cell division rate than the non-sticky cells? In order to answer this question, we have to examine the population dynamics. There is a migratory asymmetry between the liquid and surface, while cells in the liquid can only migrate to the surface when finding a vacant position, cells from the surface can always migrate to the liquid and furthermore dislodge cells to the liquid during cell division. The migration rate of cells to the liquid is therefore much higher than that of cells to the surface. The migratory asymmetry is even bigger when the fraction of sticky cells on the surface is low, because this offers fewer possibilities for non-sticky cells to adhere to the surface. The surplus of migrants to the liquid results in a much higher competitive pressure in the liquid than on the surface. Cells therefore profit if they can increase the probability of surface attachment. Sticky cells are more effective migrants than non-sticky cells. As a result, cells in the decision-making strategy evolved such that they express the sticky phenotype in the liquid. Not all the replicate simulations evolved a high fraction of sticky cells in the liquid, because mutations that trigger cell differentiation in the liquid are often harmful on the surface (S3 Fig). The pure and probabilistic strategies do not have a high fraction of sticky cells in the liquid, because cells cannot adjust their behaviour with respect to the environment in which they occur.

Another surprising result in the decision-making strategy occurs at high costs of being sticky. At *R* = 0, there are almost no sticky cells in the liquid, while approximately 20% of the cells on the surface are sticky (see black arrows in Fig 3). The lack of sticky cells in the liquid is surprising for two reasons. First, non-sticky cells cannot colonize the surface by themselves. If we would initiate our simulations with this evolved genotype, it would not be able to colonize the surface. Second, the surface contained around 2000 sticky cells. How can the number of sticky cells be so high, while sticky cells cannot divide and there are no sticky cells that migrate from the liquid to the surface? In the next section, we address the above questions by examining the decision-making strategy in more detail.

### Colony expansion and division of labour

**Fig 4:**
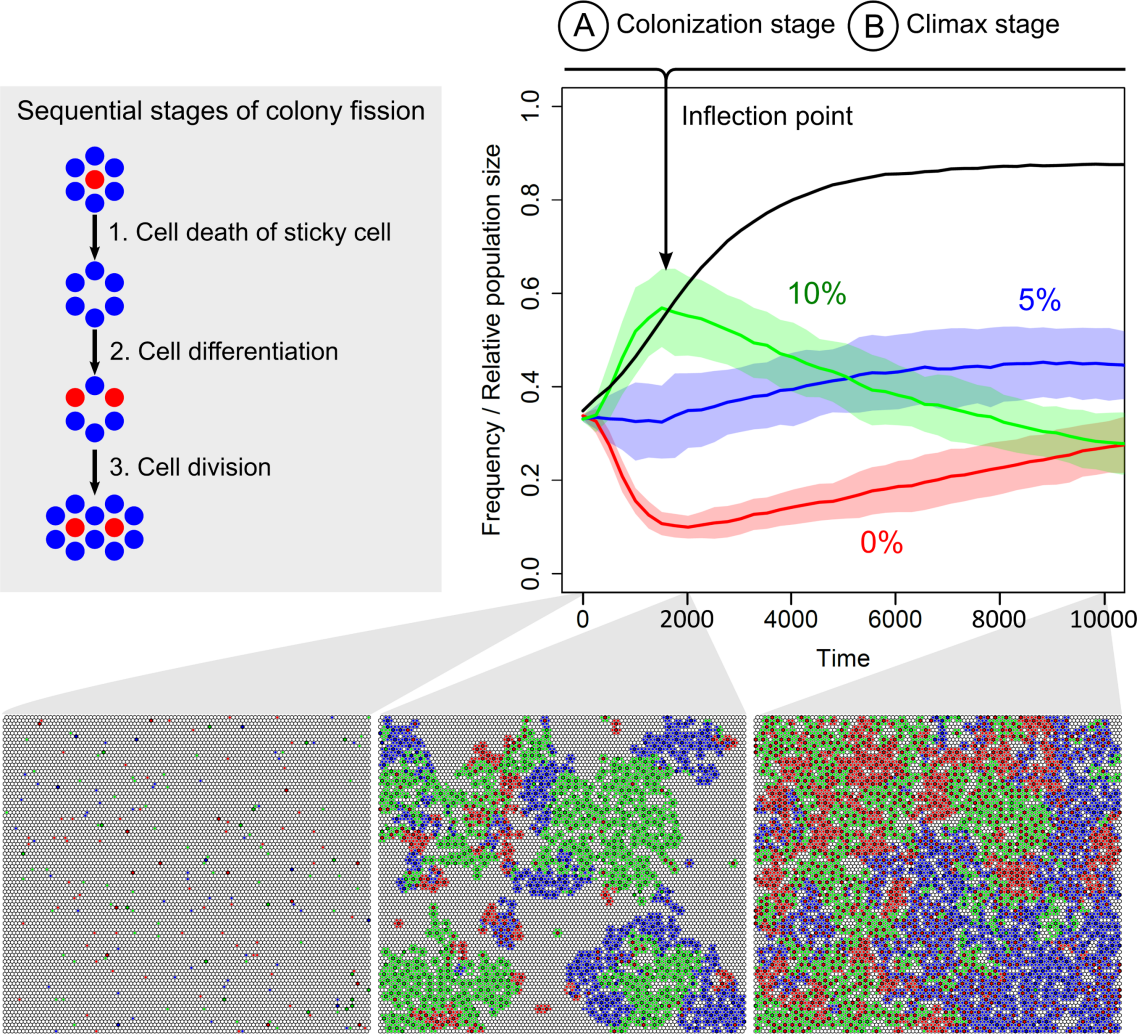
Colony expansion and cell death. (Left) Scheme showing the sequential stages of colony fission: (1) cell death, (2) cell differentiation, (3) cell division. Red and blue cells are sticky and non-sticky cells, respectively. (Right) Competition between three genotypes that differ in the death rate of sticky cells: 0% (red line), 5% (blue line) or 10% (green line) chance of cell death. Rate of cell death of non-sticky cells is the same for all genotypes (*P*_*d*_ = 10%). The average frequency ± SD (n = 10) is shown for each genotype over 10.000 time steps. The average population size (black line) is shown with respect to the total carrying capacity (including surface and liquid). At onset of competition, the population in the liquid is saturated (5.000 cells) and on the surface 100 cells of each genotype are randomly placed. Population growth is characterized by two phases: (A) population growth before the inflection point (i.e. colonization stage) and (B) population growth after the inflection point (i.e. climax stage). (Lower) Representative surfaces at different stages of competition. Colours correspond to the different genotypes (red = 0% death rate, blue = 5% death rate, green = 10% death rate). The cells with grey and black outline are non-sticky and sticky cells, respectively. Simulations were performed under low migration rate from the liquid to the surface (*P*_*m*_ = 0.01).

As shown by Fig 2, in the decision-making strategy, at *R* = 0, sticky cells are only surrounded by non-sticky neighbours. That means that a cell only differentiates when it has no sticky neighbours. If one of the neighbours is already sticky, a cell would remain non-sticky. Given this differentiation program, a colony would only be able to expand when the following sequence of fortunate events occurs (see scheme in Fig 4). First, the sticky cell should die. Second, in response to its death, some of the non-sticky neighbours should become sticky. At least two cells need to differentiate for the colony to split in two. Moreover, this should happen before the cells are dislodge from the surface, while in the absence of a sticky cell, non-sticky cells cannot stay on the surface. Finally, the remaining non-sticky neighbours have to divide in order to fill up the vacant positions next to the two novel sticky cells.

Since colony fission starts with cell death, higher death rates of the sticky cells should increase the probability of colony fission. We examine this by competing three genotypes that have the same decision-making strategy, but differ with respect to the death rate of the sticky cells: the sticky cells have a 0%, 5% or 10% probability to die, respectively (in the evolutionary simulations we assumed a 10% death probability). Hundred cells of each genotype were randomly placed on the surface. These genotypes were competed for 10.000 time steps under a reduced migration rate (*P*_*m*_ = 0.01), in order to focus on colony expansion. Fig 4 shows the frequency of each genotype over time. The genotype with the highest death rate indeed expanded faster (green line in Fig 4). This genotype had a competitive advantage over the other two genotypes at the onset of colony growth. However, when the population size went through its inflection point, the competitive advantage disappeared (see black line in Fig 4). At the inflection point, population growth is curtailed by the high cell density. There is less space to expand. As a consequence, colony longevity becomes more important for competition than colony expansion. Since lower death rates increase the longevity of a colony, the genotype with the lowest death rate slowly takes over the population of sticky cells (see S2 Fig). Once all sticky cells belong to this genotype, there is no selective difference between the genotypes anymore, because the non-sticky cells of all genotypes are identical (i.e. same fitness; see S2 Fig).

In summary, Fig 4 illustrates that a single sticky cell, together with its non-sticky neighbours, can colonize the entire surface. The sticky cell and its neighbours often have the same genotype (see time step 2000 in Fig 4), because the non-sticky cells are produced by the sticky cell before cell differentiation or *vice versa*. Thus, cells inside the colony divide labour: sticky cells sacrifice their fitness, thereby increasing the fitness of their non-sticky clonal neighbours.

### Colony expansion and ecological succession

Surface colonization in Fig 4 was characterized by two successional stages: the colonization stage and the climax stage. The colonizing genotype is favoured at low cell densities and the climax genotype at high cell densities. The successional stages in Fig 4 were based on differences in cell death (note that in the evolutionary simulations the rate of cell death could not evolve and was kept constant). Similar successional stages might as well occur for different decision-making strategies. For example, in Fig 2 we observed two distinct decision-making strategies, each associated with a unique spatial pattern. One type consisted of isolated islands of sticky cells (*R* = 0 and *R* = 0.4) and the other one of small filaments of sticky cells (*R* = 0.8). The isolated sticky cells were dominant at low *R* values and the filaments at high *R* values (Figs 2 and 3). Although the first colony type can expand over the surface, as shown in the previous section (Fig 4), there is still a substantial risk that all non-sticky cells are dislodged after the sticky cell dies. This risk is not present for the second colony type, because there are multiple concatenated sticky cells. Therefore we expected filamentous genotypes to be better colonizers.

**Fig 5:**
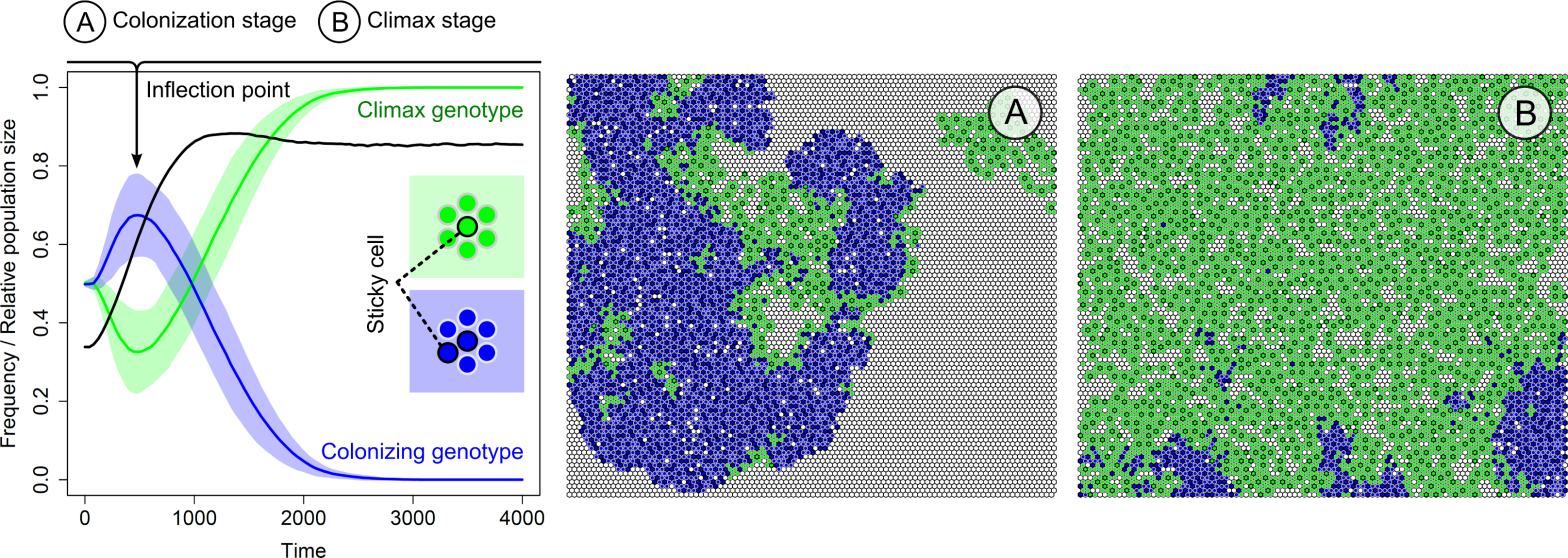
Colony expansion and filament formation. Competition between a filament-forming genotype (i.e. colonizing genotype) and a genotype that produces isolated sticky cells (i.e. climax genotype). (Left) Average frequency ± SD (n = 10) of genotypes (blue = colonizing genotype, green = climax genotype) during competition for 4.000 time steps. The average population size (black line) is shown with respect to the total carrying capacity (including surface and liquid). At onset of competition, the population in the liquid is saturated (5.000 cells) and on the surface 100 cells of each genotype are randomly placed. Population growth is characterized by two phases: (A) population growth before the inflection point (i.e. colonization stage) and (B) population growth after the inflection point (i.e. climax stage). (Right) Surfaces (A) and (B) show colony during colony expansion and at carrying capacity (for time sequence see S4 Fig). Colours correspond to the different genotypes (blue = colonizing genotype, green = climax genotype). The cells with grey and black outline are non-sticky and sticky cells, respectively. Simulations were performed under low migration rate from liquid to the surface (*P*_*m*_ = 0.01).

As in the previous section, we performed a competition experiment, placing hundred cells from each genotype–the isolated sticky cells and sticky filaments–on the surface. In order to focus on colony expansion, we reduced the migration rate from the liquid to the surface (*P*_*m*_ = 0.01). In addition, we assumed that sticky cells could not divide (*R* = 0). Fig 5 shows that filamentous sticky cells indeed function as a colonizing genotype, while the isolated sticky cells function as a climax genotype. The colonizing genotype shows a higher expansion rate than the climax genotype (S4 Fig). However, when the population size goes through its inflection point, the fitness of the colonizing genotype drops and the climax genotype takes over. The climax genotype is less efficient in colonization, but–due to the lower fraction of sticky cells–has a higher cell division rate and therefore produces more propagules that can migrate to the liquid and initiate new colonies. In the evolutionary simulations, the climax genotype dominates the surface when the costs of being sticky are high (i.e. low R values). Only when the costs of being sticky are fairly low (*R* = 0.8 in Fig 2 and Fig 3), the colonizing genotype (i.e. filamentous genotype) can outcompete the climax genotype, because the benefit of colony expansion outweigh the loss of propagule production. Since the colonizing genotype is more effective in colonizing the surface, the cell density at the surface is higher at low costs (i.e. there is a sudden increase in the cell density around R = 0.7 in Fig 3A, which corresponds to the dominance of the colonizing genotype).

Fig 5 illustrates that there is a trade-off between colony expansion and propagule production in our model: isolated sticky cells produce many propagules and expand slowly, while sticky filaments produce few propagules and expand rapidly. As a consequence, genotypes can specialize to grow at different stages of ecological succession. The colony’s expansion rate, longevity and propagule production depend on spatial pattern formation and, hence, the cell differentiation program that underlies phenotypic heterogeneity.

### Evolution of life cycles

In the previous sections, we investigated how the relative growth rate of sticky cells (*R*) affects the evolution of phenotypic heterogeneity. One would expect that ecological parameters play an important role as well. In this section, we vary both the relative cell division rate of sticky cells (*R*) and the migration rate towards the surface (*P*_*m*_). Note that migration towards the surface does not guarantee attachment, because cells first have to find an available spot, before they can actually attach. For each parameter combination we examine the evolved populations of all three differentiation strategies.

**Fig 6:**
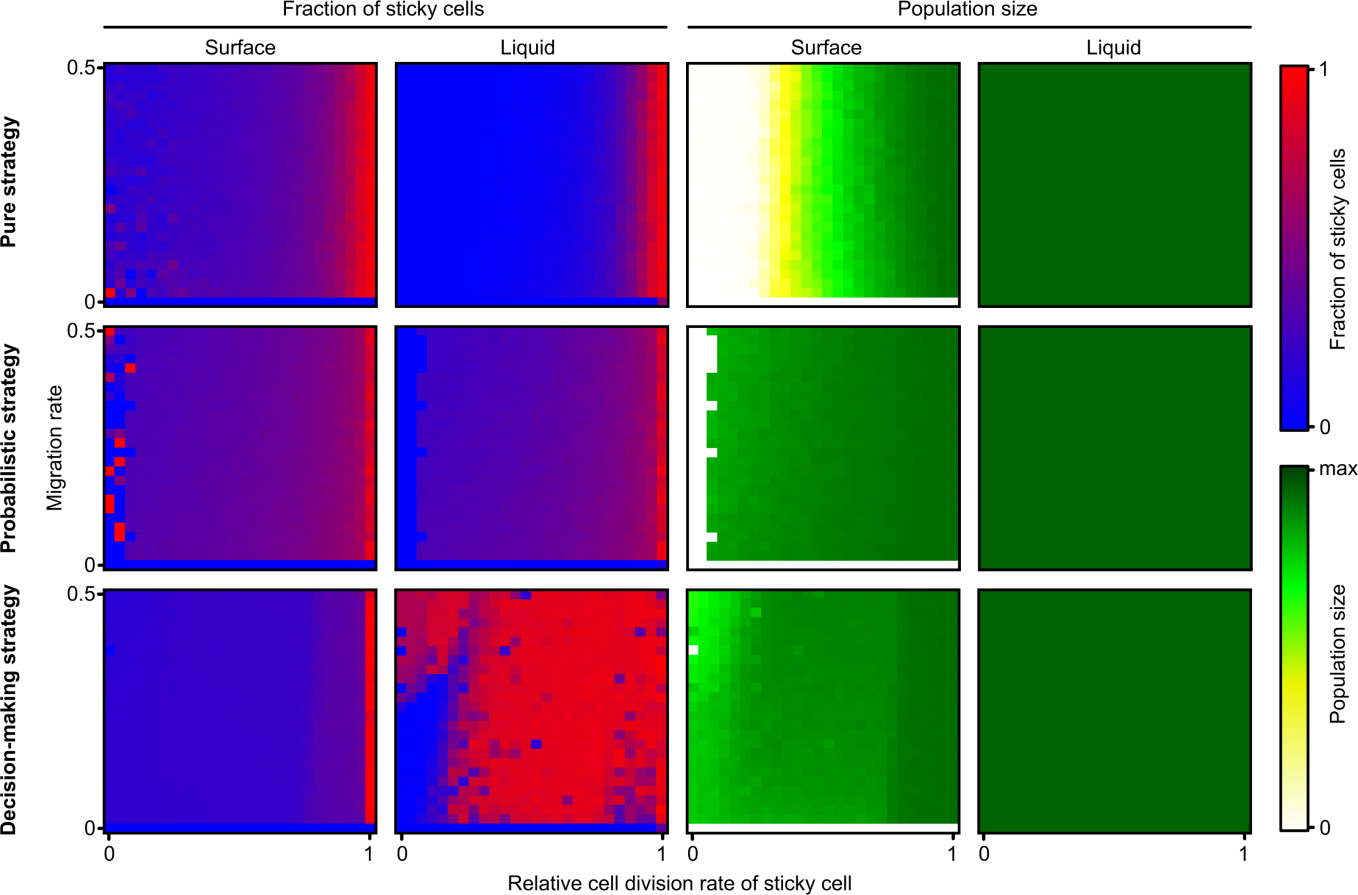
Fraction of sticky cells and population size at end of evolution. Level plots show the fraction of sticky cells and population size on the surface and in liquid for different parameter combinations of *R* (cell division rate of sticky cell) and *P*_*m*_ (migration rate). Each row of plots corresponds to one differentiation strategy. From left to right the level plots show (1) fraction of sticky cells on surface, (2) fraction of sticky cells in liquid, (3) population size on surface, (4) population size in liquid. Fraction of sticky cells ranges from 0 (blue) to 1 (red). The population size ranges from 0 (white) to the carrying capacity (green). The carrying capacity of the surface is 10.000 (100 x 100 positions on the hexagonal grid) and the carrying capacity of the liquid is 5.000 (*K*). All values are determined at the end of evolution (*T* = 400.000 time steps).

We first examined the population size and fraction of sticky cells for each parameter combination of *R* and *P*_*m*_ (*R* = 0–1 and *P*_*m*_ = 0–0.5). This was done in both the liquid and on the surface. In the pure and probabilistic strategy, the population size and fraction of sticky cells were more or less independent of the migration rate in both the liquid and on the surface (Fig 6). As shown by Fig 3, both the population size and the fraction of sticky cells decreased with the costs of being sticky (Fig 6). In the decision-making strategy, there was an effect of the migration rate, but only at high costs of being sticky. At *R* < 0.2, *higher* migration rates towards the surface paradoxically lead to *lower* population densities on the surface (Fig 6). At the same time, there is also a change in the fraction of sticky cells in the liquid. At low migration rates the fraction of sticky cells in the liquid is nearly zero, while at high migration rates it is almost one. Sticky cells are more likely to colonize the surface than non-sticky cells, which makes it even more surprising that high migration rates result in a drop of the population density on the surface. How can we explain these paradoxical results? One possible explanation for the low population density at high migration rates is the occurrence of exploitation. At high migration rates, non-sticky cells are more likely to migrate to the surface and exploit sticky cells. Such exploitation can lead to the collapse of colonies. To investigate whether or not such exploitation indeed occurs, we examined the evolutionary outcome of the decision-making strategy in more detail.

**Fig 7:**
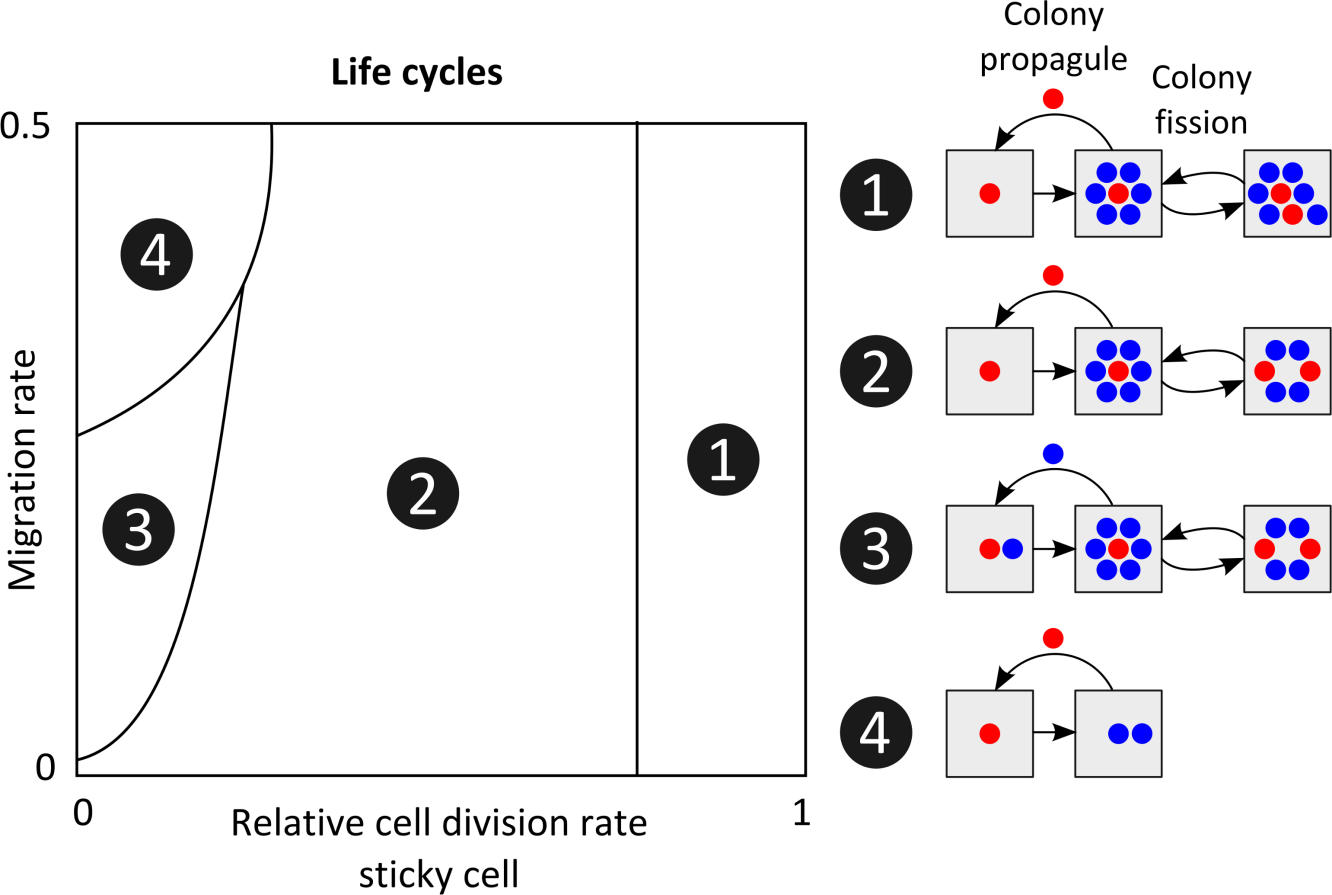
Life cycles. Schematic overview of life cycles that evolve at different relative cell division rates of sticky cells (*R*) and migration rates (*P*_*m*_). Four distinct life cycles evolved. Colonies reproduce either by producing propagules–that migrate via the liquid to new surface areas–or by fission. (Left) Parameter combinations at which different life cycles evolved (see also S5 Fig). (Right) Schematic representations of the different life cycles. Colony propagule shows behaviour of cells in the liquid, cells either remain non-sticky (blue) or differentiate to sticky cell (red). Grey squares represent surface: left squares shows the initial stage of surface colonization; the squares in the middle show the typical colony; the squares on the right show colony fission.

For each parameter combination (*R* = 0–1 and *P*_*m*_ = 0–0.5), we examined the 25 most abundant genotypes that were present at the end of evolution. The decision-making strategy was determined for each genotype. That is, we determined for which conditions a cell would differentiate to a sticky cell on the surface and for which conditions it would differentiate in the liquid. On the surface, there are eight potential differentiation strategies (S5 Fig): a cell could never or always differentiate or it could differentiate depending on the number of sticky neighbours (with 6 potential thresholds). Each parameter combination was dominated by a particular decision-making strategy that was associated with a particular life cycle (S5 Fig). Fig 7 shows the four dominant life cycles that evolved in the presence of phenotypic heterogeneity (we ignored *R* = 1 and *P*_*m*_ = 0, in which there was no heterogeneity). The life cycles consist of two stages: the colony stage at the surface and the propagule stage in the liquid. Spatial pattern formation influenced the colony properties and temporal pattern formation determined the colony’s life cycle. Colonies could reproduce in two ways: propagule production and colony fission.

In *life cycle 1*, cells formed filamentous colonies, like those shown above (Figs 2 and 7 and S6 Fig). Filaments allow for efficient colony expansion. Colonies reproduce by filament breakage, mediated by cell death or cell differentiation (i.e. colony fission), which results in two or more isolated filaments of sticky cells. At the same time, colonies reproduce by propagule production. Propagules get dislodged from the surface and migrate to establish new colonies in other regions of the surface. In *life cycle 2*, at lower *R* values, colonies lose their filamentous property (Figs 7 and S6). The costs of being sticky outweigh the advantage of being filamentous. At this stage, the colonies consist of isolated sticky cells. Even though these colonies can still expand, via cell death (Fig 4), they expand slower than the filamentous colonies. The unoccupied parts of the surface leave more space for migrating propagules to establish new colonies (Figs 6 and S6). In *life cycle 3*, at very low *R* values, there is a change in the propagule stage of the life cycle. Colonies still reproduce through fission, but propagules do not differentiate to sticky cells. Instead, they remain non-sticky and colonize the surface by exploiting the sticky cells. The non-sticky cells migrate to vacant positions that are available in existing colonies, such position are more common at low *R* values. Thus, the non-sticky migrants act as parasites that, in some cases, even take over established colonies (see S3B Fig that shows how parasitizing propagules invade in the population). In *life cycle 4*, at higher migration rates, colony formation becomes less common (S6 Fig). At high migration rates, sticky cells on the surface are more likely to be exploited by non-sticky migrants from the liquid. This exploitation breaks down the benefits of colony formation. As a consequence, a new unicellular life cycle evolves. Cells differentiate to sticky cells in the liquid, which allows for surface colonization. Once at the surface, these sticky cells de-differentiate to non-sticky cells, which can divide before being dislodged to the liquid. In this life cycle, sticky-cells can still be exploited by non-sticky migrants, but it is less likely to occur, because cells are only sticky for a transient life stage. In the absence of multicellular colony formation, there is no colony expansion and, hence, a lower cell density on the surface. This explains why higher migration rates result in lower cell densities (Fig 6).

In summary, at low costs of being sticky, colonies form filaments and reproduce by colony fission and propagule production (Fig 7). At intermediate costs, filamentous growth disappears and more space becomes available for surface colonization of propagules. At low costs, these propagules can only colonize the surface by parasitizing already existing colonies. At low costs and high migration rates, colony reproduction fails, because colonies succumb under the parasite pressure. In this case, a unicellular life cycle evolves in which surface attachment forms a transient life stage. Interestingly, the same life cycles also evolve for alternative surface geometries (e.g. triangular and square grid, instead of hexagonal grid) and for a three dimensional implementation of the surface (see S2 Text and S7–S12 Figs). Only, when a cell’s neighbourhood is discontinuous–meaning that a cell’s neighbours do not neighbour each other–surface colonization becomes impossible at low *R* values (see S2 Text).

## Discussion

Inside colonies, bacterial cells often express many different phenotypes. Using individual-based simulations, we studied the evolution of a sticky cell type in the context of surface colonization. We show that under the majority of parameter conditions surface colonization evolves. In many cases, colonization is associated with phenotypic heterogeneity, in which sticky and non-sticky cells co-occur on the surface. Phenotypic heterogeneity results from the trade-off between cell division and surface attachment (see also [50–54]): sticky cells have a reduced cell division rate, but can colonize the surface, while non-sticky cells have a high cell division rate, but cannot colonize the surface by themselves. In our model, we compared three alternative differentiation strategies: pure strategy, probabilistic strategy and decision-making strategy. In the pure strategy, cells consistently express the same phenotype and can only switch via mutations. In the probabilistic strategy, cells differentiate with a certain probability. In the decision-making strategy, cells differentiate in response to the environment. Both the probabilistic and decision-making strategy evolve surface colonization for relatively high costs of being sticky, but only the latter can colonize the surface for extreme costs–i.e. when sticky cells hardly divide (*R* << *P*_*d*_). In the decision-making strategy, cells cooperate by dividing labour: the sticky cell sacrifices its fitness for the benefit of the colony (i.e. non-sticky cells that surround the sticky cell). Cells in the probabilistic strategy can reciprocate benefits, but are not capable of coordinating their behaviour.

One striking outcome of the model is that–under the decision-making strategy–different life cycles evolved [55]. The evolution of life cycles follows from the dynamical environment in which bacterial cells live, in which they can alternate between growing on the surface and growing in the liquid. In most life cycles, the surface-attached colony forms the dominant life stage. The surface provides a scaffold on which cells can organize themselves [56,57]. The propagules in the liquid have only a marginal chance to colonize the surface. Yet, once a propagule attaches to the surface, it can form a colony that is relatively long lived and thereby produces many new propagules. Cells can affect the colony properties by coordinating their behaviour. The fitness of a colony is determined by three key properties [55]: (1) colony expansion, (2) colony longevity and (3) propagule production. Cells increase the longevity of colonies by regulating cell differentiation: if the sticky cell dies one or more neighbouring cells will differentiate, thereby guaranteeing the survival of the colony. This simple type of coordination allows for surface colonization in the toughest conditions (*R* = 0). Life cycles can *only* evolve in the decision-making strategy, because spatial and temporal organization can only come about when cells can respond to the environment [58–61]. For simplicity, we assumed that cells could only sense two environmental cues, yet, in reality cells can sense many more cues [62], which presumably allow for many alternative forms of coordination. It would be interesting to explore how environmental information facilitates or constrains spatial and temporal organization.

The surface-attached colony is vulnerable to exploitation. Cells inside the colony cooperate. As explained above, the sticky cell sacrifices its fitness for the benefit of the colony. As long as the sticky cell is surrounded by its non-sticky siblings, it cannot be exploited. However, when one of the neighbours dies, there is a risk that the cell from the liquid migrates next to the sticky cells and reaps the benefits of surface attachment without paying the costs. In our model, exploitation is more likely to occur when migration rate is high, when a cell’s neighbourhood is large and when a cell’s neighbourhood is discontinuous (see S2 Text). More generally, one could say that adhesive cells are less likely to be exploited when the diffusion of adhesive molecules is limited, such that only sibling cells profit from adhesion and invasion from outside is minimized [34,35,38,39,63–67]. A recent study of Nadell and colleagues [68] also illustrates that cells can actively prevent exploitation. They show that in *Vibrio cholerae* colonies, cells secrete a protein that facilitates a closer association between cells and the extracellular matrix. This prevents open spaces in the colony, which subsequently prevents cells from invading the interior of the colony and hence exploitation. Alternatively, non-adhesive cells might simply be less likely to join a colony than adhesive cells [69,70]. This results in the segregation of adhesive and non-adhesive cells, which in turn prevents exploitation.

Bacterial life cycles are often hard to study empirically. First, it is nearly impossible to trace bacterial individuals in nature. Second, in many cases, the ecological relevant unit of a bacterial life cycle is not the individual cell, but the colony [58,71]. Cells are relatively short-lived an only survive a part of colony formation, yet colonies often go through coordinated life stages [1,2,72]. Thus, instead of tracing the fate of a single cell, one should trace the fate of all cells in the colony. Despite these difficulties, life cycles are well characterized for a number of bacterial species [73]. For many of them, the life cycle consists of a surface-attached life stage and a unicellular dispersal stage [61,74]. In the surface-attached life stage, cells often organize into colony structures that facilitate colony expansion or dispersal. For example, many bacteria develop filamentous structures to facilitate colony expansion [75–78], while other bacteria develop fruiting bodies to facilitate dispersal [79–84]. Our study indicates that the ecological significance of the observed colony structures–and the associated adhesive cell types–can only be fully appreciated when considering the entire life cycle of a bacterium, including the dynamical environment in which bacteria make their living.

## Materials and Methods

### Experiments

Cells were grown in 2.5mL static liquid MSgg [85] using twelve-well plates. Plates were incubated for 50 hours at 30°C. The inoculum was prepared by growing strains overnight on 1.5% agar LB plates at 37°C. Overnight colonies were scraped from the plates and diluted in phosphate buffered saline (PBS) to an optical density of 0.2 (OD_600_ = 0.2). The wells were inoculated with 2μL of this sample. All strains were derived from a non-domesticated wild type *B*. *subtilis* strain called NCIB 3610 [86]. The regulatory mutant strain could not express two operons, *eps* and *tapA*, which are essential for matrix production (strain DS91, see [85,87]). The fluorescent strain expresses cyan fluorescent protein (CFP, artificially coloured red in S1 Fig) in all matrix-producing cells (strain DL823, see [16]). For microscopy, cells were isolated from top of the pellicle (i.e. colony at air-liquid interface), placed on an object glass with solidified 200μL of 1.5% agarose PBS and examined using an inverted microscope. The inverted microscope was a Nikon Eclipse TE2000-U microscope equipped with a 20× Plan Apo objective and a 60× Plan Apo oil objective. Images were taken using CFP filter. Image analysis was performed with ImageJ (1.48v).

### Model

In the model we assume there are two niches: the surface and the liquid. At the onset of evolution cells only occur in the liquid, where cells are assumed to freely float around and there is no spatial structure. The surface consists of a hexagonal grid (Fig 1). In order to colonize the surface, cells have to become ‘sticky’. When cells are sticky they can attach to the surface. Sticky cells not only facilitate their own attachment to the surface, they can also mediate non-sticky cells to adhere to the surface, but only if these non-sticky cells are located immediately adjacent to the sticky cells. Since the surface consists of a hexagonal grid, one-sticky cell can be surrounded by maximally six non-sticky cells. Stickiness typically results from the production of costly substances, such as extracellular polysaccharides, we therefore assume that being sticky reduces the rate of cell division (*R*). Yet, despite these costs, becoming sticky can be beneficial, while cells can avoid competition in the liquid by adhering to the surface. Once adhered to the surface, a cell can also migrate back to the liquid, by de-differentiating to a non-sticky cell.

At each time step, one out of five events can occur: (1) cell migration from the liquid to the surface, (2) cell migration from the surface to the liquid, (3) cell differentiation, (4) cell death and (5) cell division. For each cell, the event that occurs is selected randomly, to randomize the order in which cell events occur. We tested three different version of the model in which the strategy underlying cell differentiation is different (Fig 1). These strategies will be discussed in detail below.

#### 1. Cell migration from the liquid to the surface

Cell migration from the liquid to the surface occurs with a certain chance (*P*_*m*_). A cell migrates to a random location on the surface, where it can only attach when two criteria are fulfilled. First, the randomly selected location on the grid should still be available (i.e. there is no cell yet that is attached to this location). Second, the cell that migrates should either be sticky or should be surrounded by a sticky cell on the surface. Only by immediately neighbouring a sticky cell, a non-sticky cell can attach to the surface. When the criteria are not fulfilled, the cell immediately returns to the liquid as if nothing happened.

#### 2. Cell migration from the surface to the liquid

Cell migration from the surface to the liquid occurs when a cell is not sticky and does not have a sticky neighbour. Cell migration therefore always occurs in the local absence of sticky cells. Due to migration, the population size in the liquid becomes bigger and can even exceed its carrying capacity (*K*). To correct for this migration flux, the population size gets normalized at the end of every time step. That is, if the population size in the liquid is bigger than the carrying capacity, we simply assume that there is a dilution process (i.e. randomly selected cells from the liquid are removed) that normalizes the population size to that of the carrying capacity. Migration towards the liquid environment can also occur during cell division on the surface, in which the daughter cell is dislodged from the surface and migrates to the liquid (see details below).

#### 3. Cell differentiation

We explore the evolution of three possible strategies that could underlie differentiation (Fig 1): (i) pure strategy, (ii) probabilistic strategy, (iii) cell-decision strategy. In the first strategy, cells can either be sticky or not. They can only switch between both states by mutations; therefore the sticky and non-sticky cells correspond to different genotypes. In the second strategy, cells are sticky with a certain chance (*P*). Thus, every time a cell gets the opportunity to differentiate it has a chance, *P*, to become/remain a sticky cell and a chance, 1-*P*, to become/remain a non-sticky cell. Sticky and non-sticky cells can therefore have the same genotype. *P* can evolve (see below for details) and therefore the relative fraction of sticky and non-sticky cells can change over evolutionary time. In the last and third strategy, cells can differentiate in response to the environment. We assume that cells can sense two environmental cues: the niche (N = 0 in the liquid and N = 1 on the surface) in which they occur and the fraction of sticky cells (S). The fraction of sticky cells is determined differently on the surface and in the liquid. On the surface, cells only sense their local environment, S is therefore determined by the fraction neighbouring locations around a cell that are occupied by sticky cells (the total number of neighbouring locations is equal to six). In the liquid, S is determined by the fraction of sticky cells in the entire population. The environmental inputs are weighted by so-called connection weights (*W*_*1*_ and *W*_*2*_). A cell differentiates when the sum of regulatory input exceeds the activation threshold: *W*_*1*_*·*N + *W*_*2*_*·*S > θ. *W*_*1*_, *W*_*2*_ and θ form the genotype of an individual and can change over evolutionary time, thereby evolving the response of cells towards the environmental conditions to which they are exposed.

#### 4. Cell death

All cells have a fixed chance of dying (*P*_*d*_) that is equal to 10%. There is no difference between the death rate on the surface and in liquid and there is also no difference between the death rate of sticky and non-sticky cells (with the exception of the competition experiment in Fig 4).

#### 5. Cell division

Sticky cells have a cell division rate that is lower than or equal to that of non-sticky cells (0 ≤ *R* ≤ 1). When *R* = 1, the cell division rate of sticky and non-sticky cells is equal. When *R* = 0, sticky cells do not divide, while non-sticky cells do. Cells in the liquid can only divide when the population size in the liquid is lower than the carrying capacity (*K*). On the surface, cell division depends on the local neighbourhood of a cell. A cell can be surrounded by at most six vacant neighbouring locations. In case a cell divides, the daughter cell either goes to one of these neighbouring locations or towards the liquid. A daughter cell could only remain on the surface when surrounded by a sticky cell or when being sticky itself. In the absence of sticky cells, a daughter cell could only go to the liquid environment. The daughter cell is equally likely to go to each of the neighbouring locations on the surface as it is likely to go to the liquid. For example, if a cell has 6 available vacant neighbouring locations, then a daughter cell could end up with a chance 1/7 at each of these neighbouring locations (i.e. chance of 6/7 to remain on the surface) and with a chance 1/7 it would migrate to the liquid. The migration of a daughter cell to the liquid resembles a cell division event in the z-direction. Since we assume colonies are flat two dimensional structures, every cell division in the z-direction results in the dislodgment of a cell from the surface to the liquid. When none of the six neighbouring locations are available and the population size in the liquid is at its carrying capacity, cells on the surface cannot divide. Even though cells on the surface and in the liquid get the same number of opportunities to divide, cells on the surface have a higher chance to divide. This is because daughter cells from surface-attached cells can end up in both niches, while daughter cells from cells in the liquid cannot. Cells in the liquid are therefore more strongly constraint by the carrying capacity than cells on the surface.

In case a cell divides, the daughter cell has a certain chance to mutate (*μ*_*r*_) each of the evolvable variables. The number of evolvable variables differs between the three strategies. In case, the mutating variable is a continuous variable (e.g. *P*, *W*_*1*_, *W*_*2*_ and θ), a value is added to the variable that is taken from a normal distribution with mean *0* and standard deviation *μ*_*s*_. All simulations ran for 400.000 time steps (*T*) unless reported differently. The carrying capacity (*K*) and grid size (*G*) are kept constant for all simulations, as well as the mutation rate (*μ*_*r*_) and size (*μ*_*s*_). See S1 Table for a list of all parameter values.

## Supporting Information

S1 Fig Colony formation and phenotypic heterogeneity in *Bacillus subtilis*. (A) Top view on wells containing wild type (WT) and mutant cells over the course of 50 hours. The mutant does not produce matrix and only grows in the liquid. The WT forms a colony at the air-liquid interface, which becomes visible after approximately 30h. (B) Side view of well with WT and mutant. (C) Phenotypic heterogeneity in WT pellicle. Two representative images at different magnification levels show undifferentiated and matrix-producing cells (i.e. ‘sticky cells’). The matrix-producing cells express CFP (artificially coloured red) and sometimes form cell chains (i.e. filaments). (D) Schematic overview of pellicle formation. Grey area shows the liquid, cells that produce matrix form the colony at the air-liquid interface (i.e. surface). In the colony there are both sticky cells and undifferentiated cells that hitch-hike on the matrix produced by the sticky cells.
(TIF)

S2 Fig Colony expansion and cell death. (A) Competition between three genotypes that differ in the death rate of sticky cells: 0% (red line), 5% (blue line) and 10% (green line) chance of cell death. Rate of cell death of non-sticky cells is the same for all genotypes (P_d_ = 10%). This figure corresponds to Fig 4 from the main text, but the dynamics are shown over period of 25.000 time steps. (B) The fraction of each genotype among the populations of sticky and non-sticky cells. At the end of competition, all sticky cells come from the genotype in which sticky cells have a 0% chance to die.
(TIF)

S3 Fig Fraction of sticky cells in the decision-making strategy. (A) Fraction of sticky cells at different cell division rates at the end of evolution (*T* = 400.000 time steps). The average ± SD (n = 24) are shown by the black line and green transparent area, respectively. The data points represent the replicate simulations. (B) The fraction of sticky cells on the surface and in the liquid over evolutionary time (at intervals of 40 time steps for *T* = 400.000). The temporal dynamics are shown for *R* = 0.0, 0.2, 0.4, 0.6, 0.8 and 1.0. Each line represents one simulation (n = 24). The arrow at *R* = 0 points out a strong decrease in the fraction of sticky cells in the liquid, which results from the evolution of the non-sticky propagules that parasitize existing colonies by binding next to the sticky cells on the surface. The variability in the fraction of sticky cells on the surface is much lower than that in the liquid.
(TIF)

S4 Fig Time lapse of colony expansion. Representative colony expansion during competition between colonizing genotype (blue) and climax genotype (green). The cells with grey and black outline are non-sticky and sticky cells, respectively. The surface is shown at intervals of 100 time steps. The simulation started with 100 cells from each genotype on the surface. However, since non-sticky cells are dislodged from the surface, the initial population size quickly drops. Only a few cells eventually manage to initiate a colony. For details on competition see caption of Fig 5.
(TIF)

S5 Fig Phenotypic strategies of most abundant genotypes. For each parameter combination of *P*_*m*_ and *R* a simulation was performed. At the end of evolution, the 25 most abundant genotypes of each simulation were examined. For each genotype the phenotypic strategies on the surface and in the liquid were determined. On the surface, we determined if a genotype would always differentiate, never differentiate or differentiate when there are less than *n* neighbouring sticky cells (n = 1, 2, 3, 4, 5 or 6). In the liquid, the genotype is examined in the same way, however instead of determining if the genotype would differentiate when there are less than *n* sticky neighbours, we determine if the genotype would differentiate when the fraction of sticky cells in the population is less than *n*/6 (since cells sense the average fraction of cells). Each phenotypic strategy corresponds to a colour as shown in the legend on the right. Since we examine each genotype in both environments, every genotype is associated with two colours, one for the strategy on the surface and one for the strategy in the liquid. The strategies of the 25 most abundant genotypes are shown by the 25 colour pixels in each quadrant (i.e. every quadrant corresponds to a parameter combination). The pixels are sorted from the most abundant genotype (upper left corner of each quadrant) to the least abundant genotype (lower right corner of each quadrant) of the 25 most abundant genotypes that are present at the end of the simulation. The large black lines that are superimposed on the quadrants demarcate the sets of parameter conditions that correspond to the different life cycles.
(TIF)

S6 Fig The surface in the four evolved life cycles. For each of the life cycles discussed in Fig 7 we show a representative surface. The parameter conditions associated for each life cycle are shown on the surface (*R* = the relative cell division rate of sticky cells and *P*_*m*_ is the migration rate from the liquid to the surface). Note that for *life cycle 4* the migration rate is increased relative to that of *life cycle 3*, but the population density on the surface decreases. This is because multicellular colonies, which are good in colonizing the surface, are disfavoured by selection.
(TIF)

S7 Fig Surface geometries. We studied four different surface geometries in which cells have three (A), four (B), six (C) and eight (D) neighbours, respectively. The surface geometries with three and four neighbours are called discontinuous, because the neighbours of a cell are not each other neighbours. The surface geometries with six and eight neighbours are called continuous, because the neighbours of a cell are each other neighbours. The surface geometries were used in a two (2D version) and three dimensional (3D version) setup. In the three dimensional setup, two cell layers are placed on top of each other (for details on the interaction structure in the 3D version see S2 Text).
(TIF)

S8 Fig Top down view of surface for different surface geometries. Snapshots of surface for the different surface geometries in both the two and three dimensional model implementation. For the three dimensional implementation, cell on the top layer are shown in darker colours and are slightly smaller, so that both cell layers remain visible. For a three dimensional snapshot see S9 Fig. Surface is shown at the end of evolution T = 400.000, for *R* = 0.5 and *P*_*m*_ = 0.3.
(TIF)

S9 Fig Bird eye view of surface for different surface geometries. Three dimensional impression of the surface for the different surface geometries. Surface is shown at the end of evolution T = 400.000, for *R* = 0.5 and *P*_*m*_ = 0.3.
(TIF)

S10 Fig Fraction of sticky cells on the surface and in the liquid. Fraction of sticky cells on surface and liquid for different parameter combinations of relative cell division rate (*R*) and migration rate (*P*_*m*_). The fraction of sticky cells varies from no sticky cells (blue) to only sticky cells (red). When there are no cells on the surface a grey square is shown. Except for the surface geometry, all parameter settings are the same as in Fig 6.
(TIF)

S11 Fig Population size on the surface and in the liquid. The population size on surface and liquid for different parameter combinations of relative cell division rate (*R*) and migration rate (*P*_*m*_). The population size ranges from 0 (white) to the carrying capacity (green). When there are no cells on the surface a grey square is shown. Except for the surface geometry, all parameter settings are the same as in Fig 6.
(TIF)

S12 Fig Phenotypic strategies of most abundant genotypes. For all surface geometries and parameter combinations–*P*_*m*_ and *R*–the 25 most abundant genotypes at the end of evolution were examined. The phenotypic strategies were determined in the same way as for S5 Fig. However, in contrast to S5 Fig, cells in the different surface geometries have different number of neighbours. To facilitate comparison, we therefore determined if a genotype would differentiate on the surface when the fraction of sticky neighbours was less than *n*/6 (n = 1, 2, 3, 4, 5 or 6) using the same colour combination as in S5 Fig. Since we examine each genotype in both environments, every genotype is associated with two colours, one for the strategy on the surface and one for the strategy in the liquid. The strategies of the 25 most abundant genotypes are shown by the 25 colour pixels in each quadrant (i.e. every quadrant corresponds to a parameter combination). The pixels are sorted from the most abundant genotype (upper left corner of each quadrant) to the least abundant genotype (lower right corner of each quadrant). The quadrants that are entirely grey correspond to the parameter combinations in which there was no surface colonization.
(TIF)

S1 Table Parameter settings. (DOCX)

S1 Text Illustration of surface colonization and phenotypic heterogeneity. (DOCX)

S2 Text Surface geometry and dimensionality. (DOCX)

## References

[1] O’Toole G, Kaplan HB, Kolter R. Biofilm formation as microbial development. Annu Rev Microbiol. 2000;54: 49–79. 11018124 10.1146/annurev.micro.54.1.49

[2] Davey ME, O’toole GA. Microbial biofilms: from ecology to molecular Genetics. Microbiol Mol Biol Rev. 2000;64: 847–867. 11104821 10.1128/mmbr.64.4.847-867.2000 PMC99016

[3] Avery SV. Microbial cell individuality and the underlying sources of heterogeneity. Nat Rev Microbiol. 2006;4: 577–587. 16845428 10.1038/nrmicro1460

[4] Stewart PS, Franklin MJ. Physiological heterogeneity in biofilms. Nat Rev Microbiol. 2008;6: 199–210. 10.1038/nrmicro1838 18264116

[5] Davidson CJ, Surette MG. Individuality in Bacteria. Annu Rev Genet. 2008;42: 253–268. 10.1146/annurev.genet.42.110807.091601 18652543

[6] Perkins TJ, Swain PS. Strategies for cellular decision-making. Mol Syst Biol. 2009;5. 10.1038/msb.2009.83 PMC2795477 19920811

[7] Ackermann M. A functional perspective on phenotypic heterogeneity in microorganisms. Nat Rev Microbiol. 2015;13: 497–508. 10.1038/nrmicro3491 26145732

[8] Boles BR, Thoendel M, Singh PK. Self-generated diversity produces 'insurance effects' in biofilm communities. Proc Natl Acad Sci U S A. 2004;101: 16630–16635. 15546998 10.1073/pnas.0407460101 PMC528905

[9] Cárcamo-Oyarce G, Lumjiaktase P, Kümmerli R, Eberl L. Quorum sensing triggers the stochastic escape of individual cells from *Pseudomonas putida* biofilms. Nat Commun. 2015;6. 10.1038/ncomms6945 PMC4309448 25592773

[10] Kearns DB, Losick R. Cell population heterogeneity during growth of *Bacillus subtilis*. Genes Dev. 2005;19: 3083–3094. 16357223 10.1101/gad.1373905 PMC1315410

[11] Chai Y, Chu F, Kolter R, Losick R. Bistability and biofilm formation in *Bacillus subtilis*. Mol Microbiol. 2008;67: 254–263. 18047568 10.1111/j.1365-2958.2007.06040.x PMC2430929

[12] Chai Y, Norman T, Kolter R, Losick R. An epigenetic switch governing daughter cell separation in *Bacillus subtilis*. Genes Dev. 2010;24: 754–765. 10.1101/gad.1915010 20351052 PMC2854391

[13] Norman TM, Lord ND, Paulsson J, Losick R. Memory and modularity in cell-fate decision making. Nature. 2013;503:481–486. 10.1038/nature12804 24256735 PMC4019345

[14] Vlamakis H, Aguilar C, Losick R, Kolter R. Control of cell fate by the formation of an architecturally complex bacterial community. Genes Dev. 2008;22: 945–953. 10.1101/gad.1645008 18381896 PMC2279205

[15] Kearns DB. Division of labour during *Bacillus subtilis* biofilm formation. Mol Microbiol. 2008;67: 229–231. 18086186 10.1111/j.1365-2958.2007.06053.x

[16] Lopez D, Vlamakis H, Losick R, Kolter R. Paracrine signaling in a bacterium. Genes Dev. 2009;23: 1631–1638. 10.1101/gad.1813709 19605685 PMC2714712

[17] Lopez D, Kolter R. Extracellular signals that define distinct and coexisting cell fates in *Bacillus subtilis*. FEMS Microbiol Rev. 2010;34: 134–149. 10.1111/j.1574-6976.2009.00199.x 20030732

[18] Ostrowski A, Mehert A, Prescott A, Kiley TB, Stanley-Wall NR. YuaB functions synergistically with the exopolysaccharide and TasA amyloid fibers to allow biofilm formation by *Bacillus subtilis*. J Bacteriol. 2011;193: 4821–4831. 10.1128/JB.00223-11 21742882 PMC3165672

[19] van Gestel J, Vlamakis H, Kolter R. From cell differentiation to cell collectives: *Bacillus subtilis* uses division of labor to migrate. PLoS Biol. 2015;13: e1002141 10.1371/journal.pbio.1002141 25894589 PMC4403855

[20] Branda SS, Vik A, Friedman L, Kolter R. Biofilms: the matrix revisited. Trends Microbiol. 2005;13: 20–26. 15639628 10.1016/j.tim.2004.11.006

[21] Flemming HC, Wingender J. The biofilm matrix. Nat Rev Microbiol. 2010;8: 623–633. 10.1038/nrmicro2415 20676145

[22] Marvasi M, Visscher PT, Martinez LC. Exopolymeric substances (EPS) from *Bacillus subtilis*: polymers and genes encoding their synthesis. FEMS Microbiol Lett. 2010;313: 1–9. 10.1111/j.1574-6968.2010.02085.x 20735481

[23] Berk V, Fong JCN, Dempsey GT, Develioglu ON, Zhuang X, Liphardt J, et al Molecular architecture and assembly principles of *Vibrio cholerae* biofilms. Science. 2012;337: 236–239. 10.1126/science.1222981 22798614 PMC3513368

[24] Rainey PB, Travisano M. Adaptive radiation in a heterogeneous environment. Nature. 1998;394: 69–72. 9665128 10.1038/27900

[25] Spiers AJ, Kahn SG, Bohannon J, Travisano M, Rainey PB. Adaptive divergence in experimental populations of *Pseudomonas fluorescens*. I. Genetic and phenotypic bases of wrinkly spreader fitness. Genetics. 2002;161: 33–46. 12019221 10.1093/genetics/161.1.33 PMC1462107

[26] Rainey PB, Rainey K. Evolution of cooperation and conflict in experimental bacterial populations. Nature. 2003;425: 72–74. 12955142 10.1038/nature01906

[27] Nadell CD, Bassler BL. A fitness trade-off between local competition and dispersal in *Vibrio cholerae* biofilms. Proc Natl Acad Sci U S A. 2011;108: 14181–14185. 10.1073/pnas.1111147108 21825170 PMC3161532

[28] Poltak SR, Cooper VS. Ecological succession in long-term experimentally evolved biofilms produces synergistic communities. ISME J. 2011;5: 369–378. 10.1038/ismej.2010.136 20811470 PMC3105725

[29] Traverse CC, Mayo-Smith LM, Poltak SR, Cooper VS. Tangled bank of experimentally evolved *Burkholderia* biofilms reflects selection during chronic infections. Proc Natl Acad Sci U S A. 2013;110: E250–E259. 10.1073/pnas.1207025110 23271804 PMC3549113

[30] Hammerschmidt K, Rose CJ, Kerr B, Rainey PB. Life cycles, fitness decoupling and the evolution of multicellularity. Nature. 2014;515: 75–79. 10.1038/nature13884 25373677

[31] Kreft JU, Picioreanu C, Wimpenny JWT, van Loosdrecht MCM. Individual-based modelling of biofilms. Microbiology. 2001;147: 2897–2912. 11700341 10.1099/00221287-147-11-2897

[32] Nadell CD, Xavier JB, Foster KR. The sociobiology of biofilms. FEMS Microbiol Rev. 2009;33: 206–224. 10.1111/j.1574-6976.2008.00150.x 19067751

[33] Hallatschek O, Hersen P, Ramanathan S, Nelson DR. Genetic drift at expanding frontiers promotes gene segregation. Proc Natl Acad Sci U S A. 2007;104: 19926–19930. 18056799 10.1073/pnas.0710150104 PMC2148399

[34] Xavier JB, Foster KR. Cooperation and conflict in microbial biofilms. Proc Natl Acad Sci U S A. 2007;104: 876–881. 17210916 10.1073/pnas.0607651104 PMC1783407

[35] Nadell CD, Foster KR, Xavier JB. Emergence of spatial structure in cell groups and the evolution of cooperation. PLoS Comput Biol. 2010;6: e1000716 10.1371/journal.pcbi.1000716 20333237 PMC2841614

[36] Korolev KS, Müller MJI, Karahan N, Murray AW, Hallatschek O, Nelson DR. Selective sweeps in growing microbial colonies. Phys Biol. 2012;9: 026008 10.1088/1478-3975/9/2/026008 22476106 PMC3359763

[37] Janulevicius A, van Loosdrecht M, Picioreanu C. Short-range guiding can result in the formation of circular aggregates in Myxobacteria Populations. PLoS Comput Biol. 2015;11: e1004213 10.1371/journal.pcbi.1004213 25928112 PMC4415783

[38] van Gestel J, Weissing FJ, Kuipers OP, Kovács ÁT. Density of founder cells affects spatial pattern formation and cooperation in *Bacillus subtilis* biofilms. ISME J. 2014;8: 2069–2079. 10.1038/ismej.2014.52 24694715 PMC4184017

[39] Schluter J, Nadell CD, Bassler BL, Foster KR. Adhesion as a weapon in microbial competition. ISME J. 2015;9: 139–149. 10.1038/ismej.2014.174 25290505 PMC4268496

[40] Solé RV, Valverde S. Before the endless forms: embodied model of transition from single cells to aggregates to ecosystem engineering. PLoS ONE. 2013;8: e59664 10.1371/journal.pone.0059664 23596506 PMC3626615

[41] Kobayashi K. *Bacillus subtilis* pellicle formation proceeds through genetically defined morphological changes. J Bacteriol. 2007;189: 4920–4931. 17468240 10.1128/JB.00157-07 PMC1913431

[42] Brückner S, Mösch HU. Choosing the right lifestyle: adhesion and development in *Saccharomyces cerevisiae*. FEMS Microbiol Rev. 2012;36: 25–58. 10.1111/j.1574-6976.2011.00275.x 21521246

[43] Lugtenberg BJ, Dekkers L, Bloemberg GV. Molecular determinants of rhizosphere colonization by *Pseudomonas*. Annu Rev Phytopathol. 2001;39: 461–490. 11701873 10.1146/annurev.phyto.39.1.461

[44] Beauregard PB, Chai Y, Vlamakis H, Losick R, Kolter R. *Bacillus subtilis* biofilm induction by plant polysaccharides. Proc Natl Acad Sci U S A. 2013;110: E1621–1630. 10.1073/pnas.1218984110 23569226 PMC3637697

[45] Bais HP, Fall R, Vivanco JM. Biocontrol of *Bacillus subtilis* against infection of Arabidopsis roots by *Pseudomonas syringae* is facilitated by biofilm formation and surfactin production. Plant Physiol. 2004;134: 307–319. 14684838 10.1104/pp.103.028712 PMC316310

[46] Chen Y, Yan F, Chai Y, Liu H, Kolter R, Losick R, et al Biocontrol of tomato wilt disease by *Bacillus subtilis* isolates from natural environments depends on conserved genes mediating biofilm formation. Environ Microbiol. 2013;15: 848–864. 10.1111/j.1462-2920.2012.02860.x 22934631 PMC3904073

[47] Young IM, Crawford JW. Interactions and self-organization in the soil-microbe complex. Science. 2004;304: 1634–1637. 15192219 10.1126/science.1097394

[48] Vilain S, Luo Y, Hildreth MB, Brozel VS. Analysis of the life cycle of the soil saprophyte *Bacillus cereus* in liquid soil extract and in soil. Appl Environ Microbiol. 2006;72: 4970–4977. 16820495 10.1128/AEM.03076-05 PMC1489341

[49] Balbontín R, Vlamakis H, Kolter R. Mutualistic interaction between *Salmonella enterica* and *Aspergillus niger* and its effects on *Zea mays* colonization. Microb Biotechnol. 2014;7: 589–600. 10.1111/1751-7915.12182 25351041 PMC4265077

[50] Johnson DR, Goldschmidt F, Lilja EE, Ackermann M. Metabolic specialization and the assembly of microbial communities. ISME J. 2012;6: 1985–1991. 10.1038/ismej.2012.46 22592822 PMC3475376

[51] Ackermann M, Stecher B, Freed NE, Songhet P, Hardt WD, Doebeli M. Self-destructive cooperation mediated by phenotypic noise. Nature. 2008;454: 987–990. 10.1038/nature07067 18719588

[52] Michod RE. The group covariance effect and fitness trade-offs during evolutionary transitions in individuality. Proc Natl Acad Sci U S A. 2006;103: 9113–9117. 16751277 10.1073/pnas.0601080103 PMC1482575

[53] Gavrilets S. Rapid transition towards the division of labor via evolution of developmental plasticity. PLoS Comput Biol. 2010;6: e1000805 10.1371/journal.pcbi.1000805 20548941 PMC2883585

[54] Ispolatov I, Ackermann M, Doebeli M. Division of labour and the evolution of multicellularity. Proc R Soc B Biol Sci. 2011;279: 1768–1776. 10.1098/rspb.2011.1999 PMC3297448 22158952

[55] McDougald D, Rice SA, Barraud N, Steinberg PD, Kjelleberg S. Should we stay or should we go: mechanisms and ecological consequences for biofilm dispersal. Nat Rev Microbiol. 2012;10: 39–50. 10.1038/nrmicro2695 22120588

[56] Wolpert L. Positional information and the spatial pattern of cellular differentiation. J Theor Biol. 1969;25: 1–47. 4390734 10.1016/s0022-5193(69)80016-0

[57] Kolter R, Greenberg EP. Microbial sciences: The superficial life of microbes. Nature. 2006;441: 300–302. 16710410 10.1038/441300a

[58] Shapiro JA. Thinking about bacterial populations as multicellular organisms. Annu Rev Microbiol. 1998;52: 81–104. 9891794 10.1146/annurev.micro.52.1.81

[59] Furusawa C, Kaneko K. Emergence of multicellular organisms with dynamic differentiation and spatial pattern. Artif Life. 1998;4: 79–93. 9798276 10.1162/106454698568459

[60] Boutte CC, Crosson S. Bacterial lifestyle shapes stringent response activation. Trends Microbiol. 2013;21: 174–180. 10.1016/j.tim.2013.01.002 23419217 PMC4238387

[61] Bonner JT. Life Cycles: Reflections of an Evolutionary Biologist. Princeton University Press; 1995.

[62] Karatan E, Watnick P. Signals, regulatory networks, and materials that build and break bacterial biofilms. Microbiol Mol Biol Rev. 2009;73: 310–347. 10.1128/MMBR.00041-08 19487730 PMC2698413

[63] Nowak MA, Bonhoeffer S, May RM. Spatial games and the maintenance of cooperation. Proc Natl Acad Sci U S A. 1994;91: 4877–4881. 8197150 10.1073/pnas.91.11.4877 PMC43892

[64] Kreft JU. Conflicts of interest in biofilms. Biofilms. 2004;1: 265–276.

[65] Nowak MA. Five rules for the evolution of cooperation. Science. 2006;314: 1560–1563. 17158317 10.1126/science.1133755 PMC3279745

[66] Allen B, Gore J, Nowak MA. Spatial dilemmas of diffusible public goods. eLife. 2013;2:e01169 10.7554/eLife.01169 24347543 PMC3865686

[67] Mitri S, Xavier JB, Foster KR. Social evolution in multispecies biofilms. Proc Natl Acad Sci U S A. 2011;108: 10839–10846. 10.1073/pnas.1100292108 21690380 PMC3131810

[68] Nadell CD, Drescher K, Wingreen NS, Bassler BL. Extracellular matrix structure governs invasion resistance in bacterial biofilms. ISME J. 2015. 10.1038/ismej.2014.246 PMC4511925 25603396

[69] Garcia T, De Monte S. Group formation and the evolution of sociality. Evolution. 2013;67: 131–141. 10.1111/j.1558-5646.2012.01739.x 23289567

[70] Garcia T, Doulcier G, Monte SD. The evolution of adhesiveness as a social adaptation. eLife. 2015; e08595 10.7554/eLife.08595 26613415 PMC4775229

[71] Shapiro JA. Bacteria as multicellular organisms. Sci Am. 1988;258: 82–89.2847312

[72] Monds RD, O’Toole GA. The developmental model of microbial biofilms: ten years of a paradigm up for review. Trends Microbiol. 2009;17: 73–87. 10.1016/j.tim.2008.11.001 19162483

[73] Shimkets DLJ. Prokaryotic Life Cycles In: Rosenberg E, DeLong EF, Lory S, Stackebrandt E, Thompson F, editors. The Prokaryotes. Springer Berlin Heidelberg; 2013 pp. 317–336.

[74] Bonner JT. First signals: the Evolution of Multicellular Development Princeton University Press; 2001.

[75] Flärdh K, Buttner MJ. *Streptomyces* morphogenetics: dissecting differentiation in a filamentous bacterium. Nat Rev Microbiol. 2009;7: 36–49. 10.1038/nrmicro1968 19079351

[76] McCormick JR, Flärdh K. Signals and regulators that govern *Streptomyces* development. FEMS Microbiol Rev. 2012;36: 206–231. 10.1111/j.1574-6976.2011.00317.x 22092088 PMC3285474

[77] Claessen D, Rozen DE, Kuipers OP, Søgaard-Andersen L, van Wezel GP. Bacterial solutions to multicellularity: a tale of biofilms, filaments and fruiting bodies. Nat Rev Microbiol. 2014;12: 115–124. 10.1038/nrmicro3178 24384602

[78] Lyons NA, Kolter R. On the evolution of bacterial multicellularity. Curr Opin Microbiol. 2015;24: 21–28. 10.1016/j.mib.2014.12.007 25597443 PMC4380822

[79] Velicer GJ, Vos M. Sociobiology of the Myxobacteria. Annu Rev Microbiol. 2009;63: 599–623. 10.1146/annurev.micro.091208.073158 19575567

[80] Konovalova A, Petters T, Sogaard-Andersen L. Extracellular biology of *Myxococcus xanthus*. FEMS Microbiol Rev. 2010;34: 89–106. 10.1111/j.1574-6976.2009.00194.x 19895646

[81] Higgs PI, Hartzell PL, Holkenbrink C, Hoiczyk E. *Myxococcus xanthus* vegetative and developmental cell heterogeneity In: Yang Z, Higgs PI, editors. Myxobacteria: Genomics, Cellular and Molecular Biology. Norfolk, UK: Horizon Scientific Press; 2014.

[82] Webb JS, Givskov M, Kjelleberg S. Bacterial biofilms: prokaryotic adventures in multicellularity. Curr Opin Microbiol. 2003;6: 578–585. 14662353 10.1016/j.mib.2003.10.014

[83] Webb JS, Thompson LS, James S, Charlton T, Tolker-Nielsen T, Koch B, et al Cell death in *Pseudomonas aeruginosa* biofilm development. J Bacteriol. 2003;185: 4585–4592. 12867469 10.1128/JB.185.15.4585-4592.2003 PMC165772

[84] Kirov SM, Webb JS, O’May CY, Reid DW, Woo JKK, Rice SA, et al Biofilm differentiation and dispersal in mucoid *Pseudomonas aeruginosa* isolates from patients with cystic fibrosis. Microbiology. 2007;153: 3264–3274. 17906126 10.1099/mic.0.2007/009092-0

[85] Branda SS, Chu F, Kearns DB, Losick R, Kolter R. A major protein component of the *Bacillus subtilis* biofilm matrix. Mol Microbiol. 2006;59: 1229–1238. 16430696 10.1111/j.1365-2958.2005.05020.x

[86] Branda SS, González-Pastor JE, Ben-Yehuda S, Losick R, Kolter R. Fruiting body formation by *Bacillus subtilis*. Proc Natl Acad Sci U S A. 2001;98: 11621–11626. 11572999 10.1073/pnas.191384198 PMC58779

[87] Kearns DB, Chu F, Branda SS, Kolter R, Losick R. A master regulator for biofilm formation by *Bacillus subtilis*. Mol Microbiol. 2005;55: 739–749. 15661000 10.1111/j.1365-2958.2004.04440.x

