# Supplementary figures and images for: Phenotypic Heterogeneity and the Evolution of Bacterial Life Cycles
Source: PLoS Comput Biol. 2016 Feb 19;12(2):e1004764. doi: 10.1371/journal.pcbi.1004764 (PMC4760940; doi:10.1371/journal.pcbi.1004764)

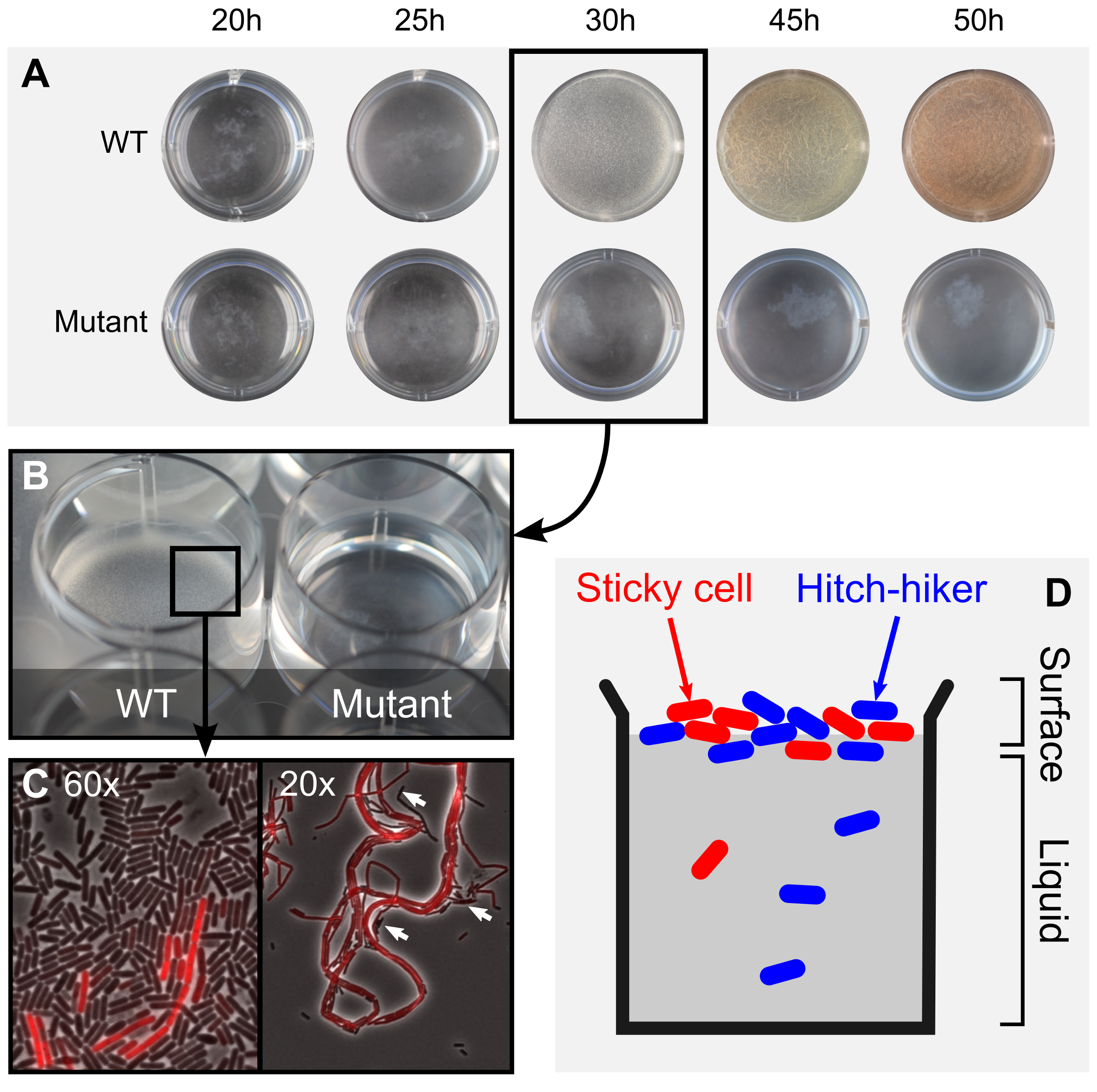

Supplement: S1 Fig — (A) Top view on wells containing wild type (WT) and mutant cells over the course of 50 hours. The mutant does not produce matrix and only grows in the liquid. The WT forms a colony at the air-liquid interface, which becomes visible after approximately 30h. (B) Side view of well with WT and mutant. (C) Phenotypic heterogeneity in WT pellicle. Two representative images at different magnification levels show undifferentiated and matrix-producing cells (i.e. ‘sticky cells’). The matrix-producing cells express CFP (artificially coloured red) and sometimes form cell chains (i.e. filaments). (D) Schematic overview of pellicle formation. Grey area shows the liquid, cells that produce matrix form the colony at the air-liquid interface (i.e. surface). In the colony there are both sticky cells and undifferentiated cells that hitch-hike on the matrix produced by the sticky cells. (TIF) [file pcbi.1004764.s001.tif]

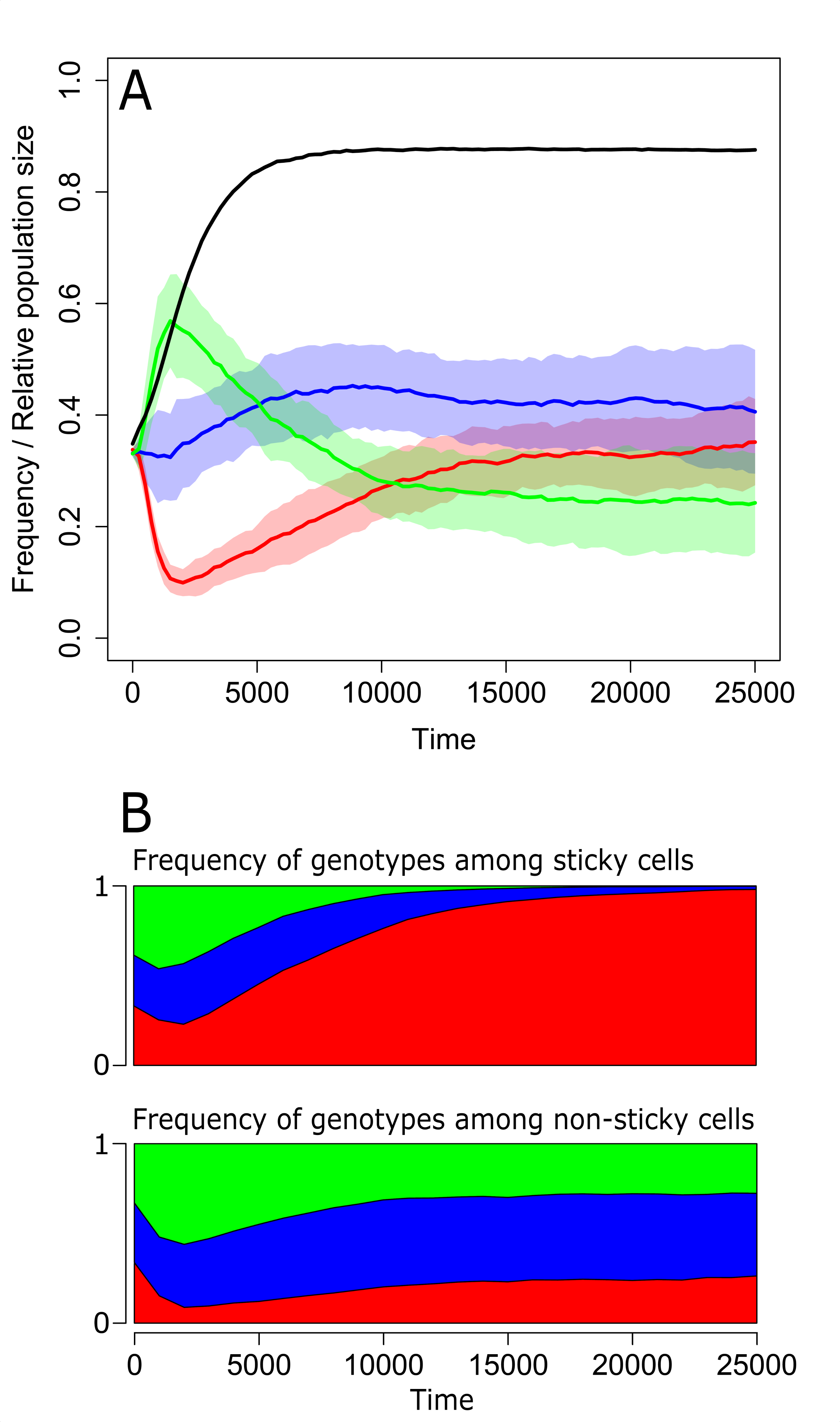

Supplement: S2 Fig — (A) Competition between three genotypes that differ in the death rate of sticky cells: 0% (red line), 5% (blue line) and 10% (green line) chance of cell death. Rate of cell death of non-sticky cells is the same for all genotypes (Pd = 10%). This figure corresponds to Fig 4 from the main text, but the dynamics are shown over period of 25.000 time steps. (B) The fraction of each genotype among the populations of sticky and non-sticky cells. At the end of competition, all sticky cells come from the genotype in which sticky cells have a 0% chance to die. (TIF) [file pcbi.1004764.s002.tif]

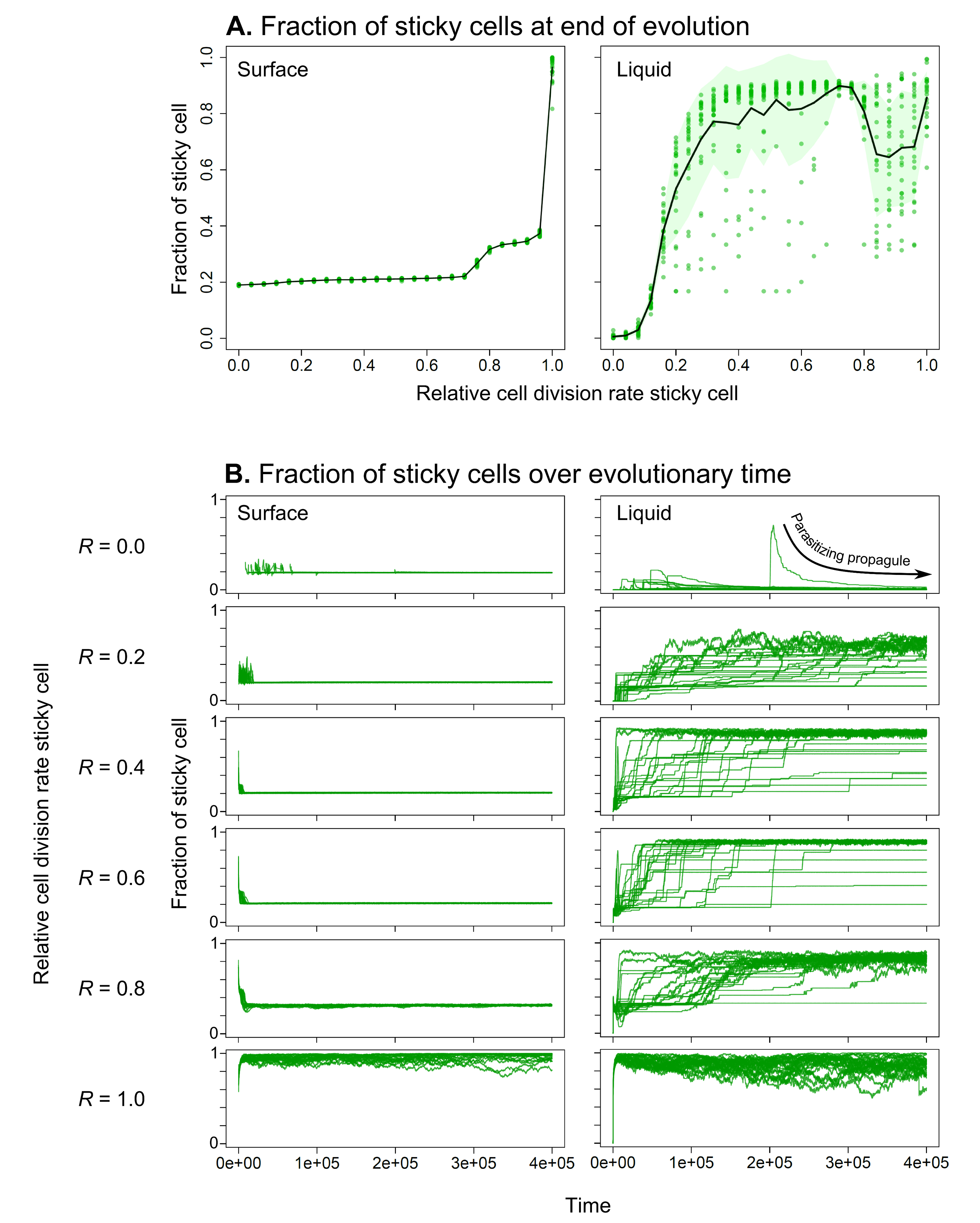

Supplement: S3 Fig — (A) Fraction of sticky cells at different cell division rates at the end of evolution (T = 400.000 time steps). The average ± SD (n = 24) are shown by the black line and green transparent area, respectively. The data points represent the replicate simulations. (B) The fraction of sticky cells on the surface and in the liquid over evolutionary time (at intervals of 40 time steps for T = 400.000). The temporal dynamics are shown for R = 0.0, 0.2, 0.4, 0.6, 0.8 and 1.0. Each line represents one simulation (n = 24). The arrow at R = 0 points out a strong decrease in the fraction of sticky cells in the liquid, which results from the evolution of the non-sticky propagules that parasitize existing colonies by binding next to the sticky cells on the surface. The variability in the fraction of sticky cells on the surface is much lower than that in the liquid. (TIF) [file pcbi.1004764.s003.tif]

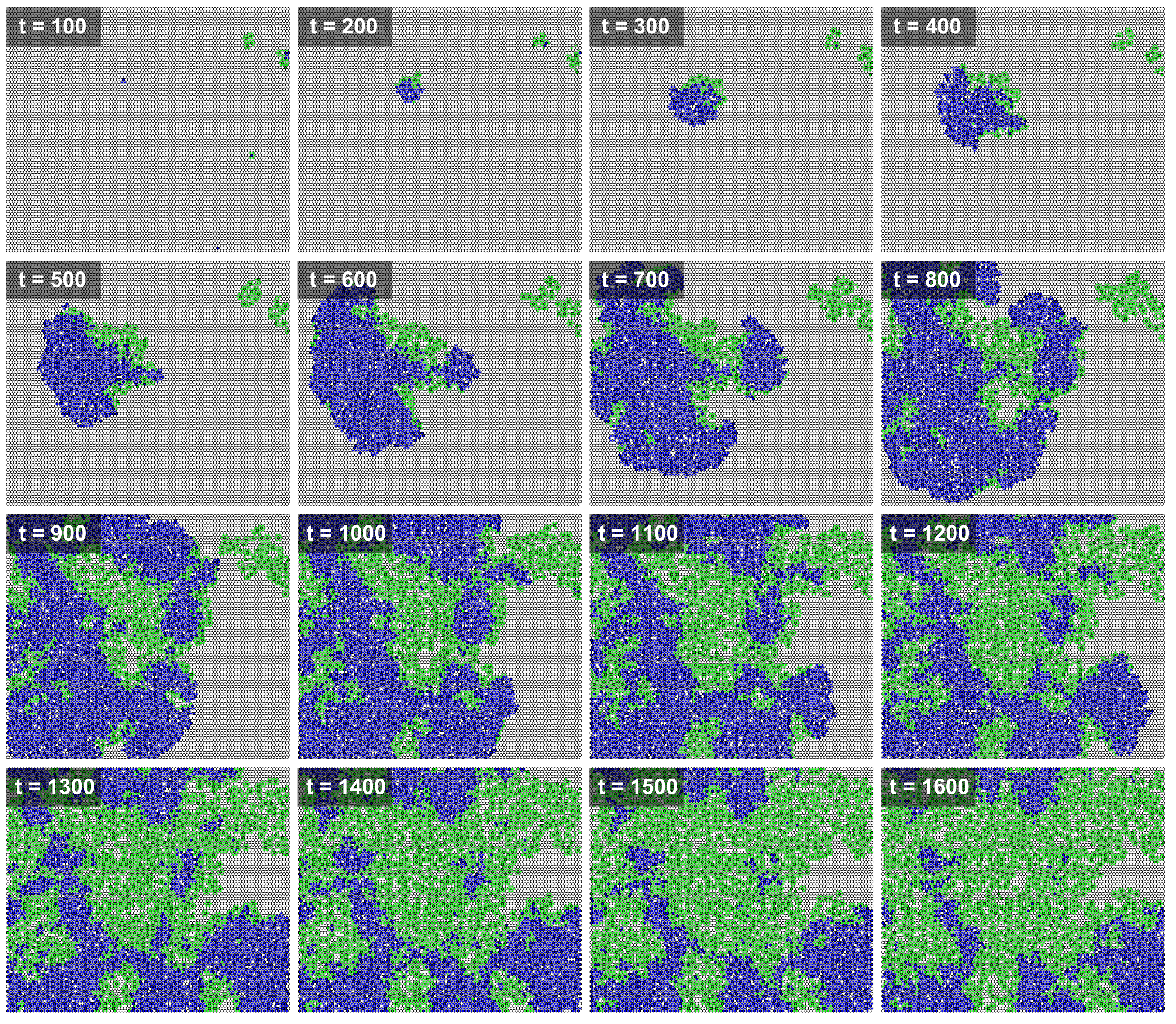

Supplement: S4 Fig — Representative colony expansion during competition between colonizing genotype (blue) and climax genotype (green). The cells with grey and black outline are non-sticky and sticky cells, respectively. The surface is shown at intervals of 100 time steps. The simulation started with 100 cells from each genotype on the surface. However, since non-sticky cells are dislodged from the surface, the initial population size quickly drops. Only a few cells eventually manage to initiate a colony. For details on competition see caption of Fig 5. (TIF) [file pcbi.1004764.s004.tif]

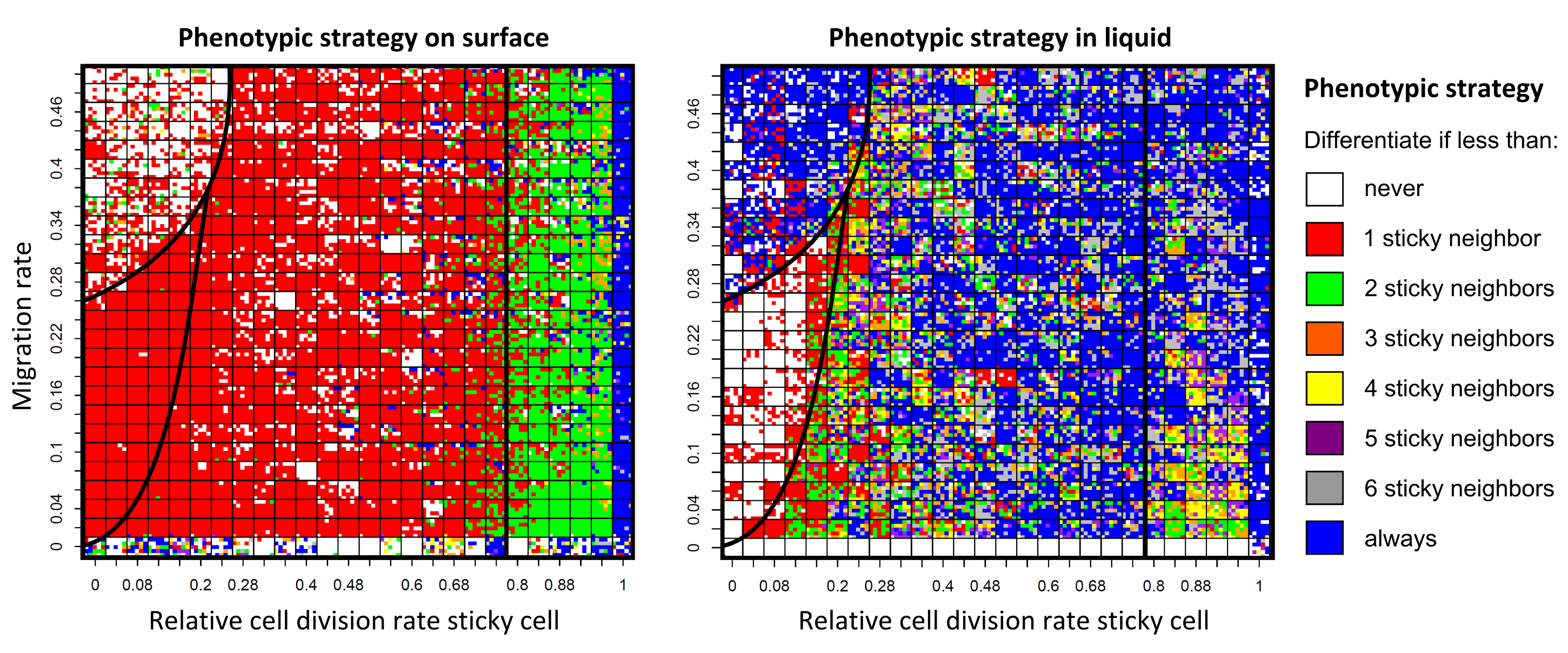

Supplement: S5 Fig — For each parameter combination of Pm and R a simulation was performed. At the end of evolution, the 25 most abundant genotypes of each simulation were examined. For each genotype the phenotypic strategies on the surface and in the liquid were determined. On the surface, we determined if a genotype would always differentiate, never differentiate or differentiate when there are less than n neighbouring sticky cells (n = 1, 2, 3, 4, 5 or 6). In the liquid, the genotype is examined in the same way, however instead of determining if the genotype would differentiate when there are less than n sticky neighbours, we determine if the genotype would differentiate when the fraction of sticky cells in the population is less than n/6 (since cells sense the average fraction of cells). Each phenotypic strategy corresponds to a colour as shown in the legend on the right. Since we examine each genotype in both environments, every genotype is associated with two colours, one for the strategy on the surface and one for the strategy in the liquid. The strategies of the 25 most abundant genotypes are shown by the 25 colour pixels in each quadrant (i.e. every quadrant corresponds to a parameter combination). The pixels are sorted from the most abundant genotype (upper left corner of each quadrant) to the least abundant genotype (lower right corner of each quadrant) of the 25 most abundant genotypes that are present at the end of the simulation. The large black lines that are superimposed on the quadrants demarcate the sets of parameter conditions that correspond to the different life cycles. (TIF) [file pcbi.1004764.s005.tif]

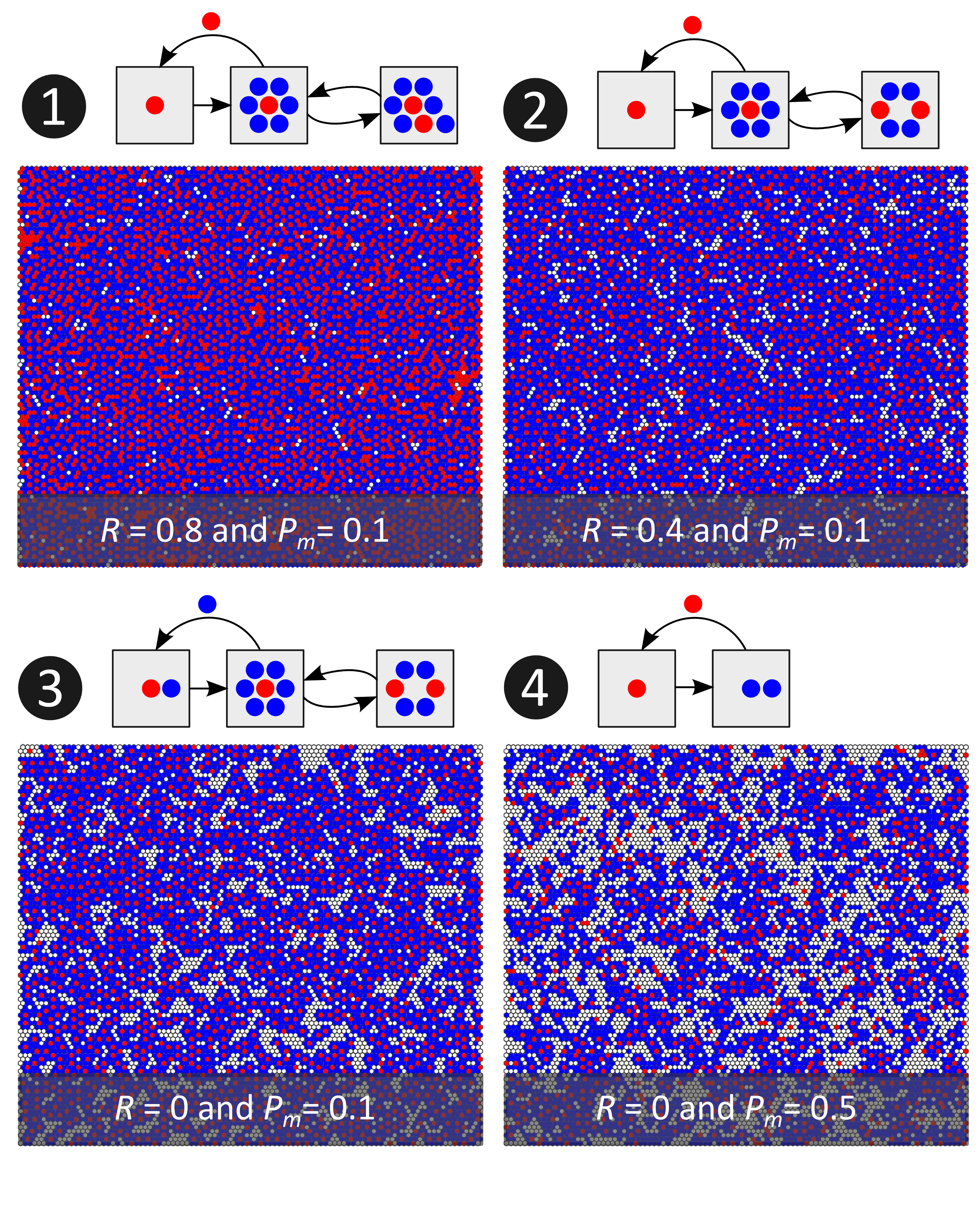

Supplement: S6 Fig — For each of the life cycles discussed in Fig 7 we show a representative surface. The parameter conditions associated for each life cycle are shown on the surface (R = the relative cell division rate of sticky cells and Pm is the migration rate from the liquid to the surface). Note that for life cycle 4 the migration rate is increased relative to that of life cycle 3, but the population density on the surface decreases. This is because multicellular colonies, which are good in colonizing the surface, are disfavoured by selection. (TIF) [file pcbi.1004764.s006.tif]

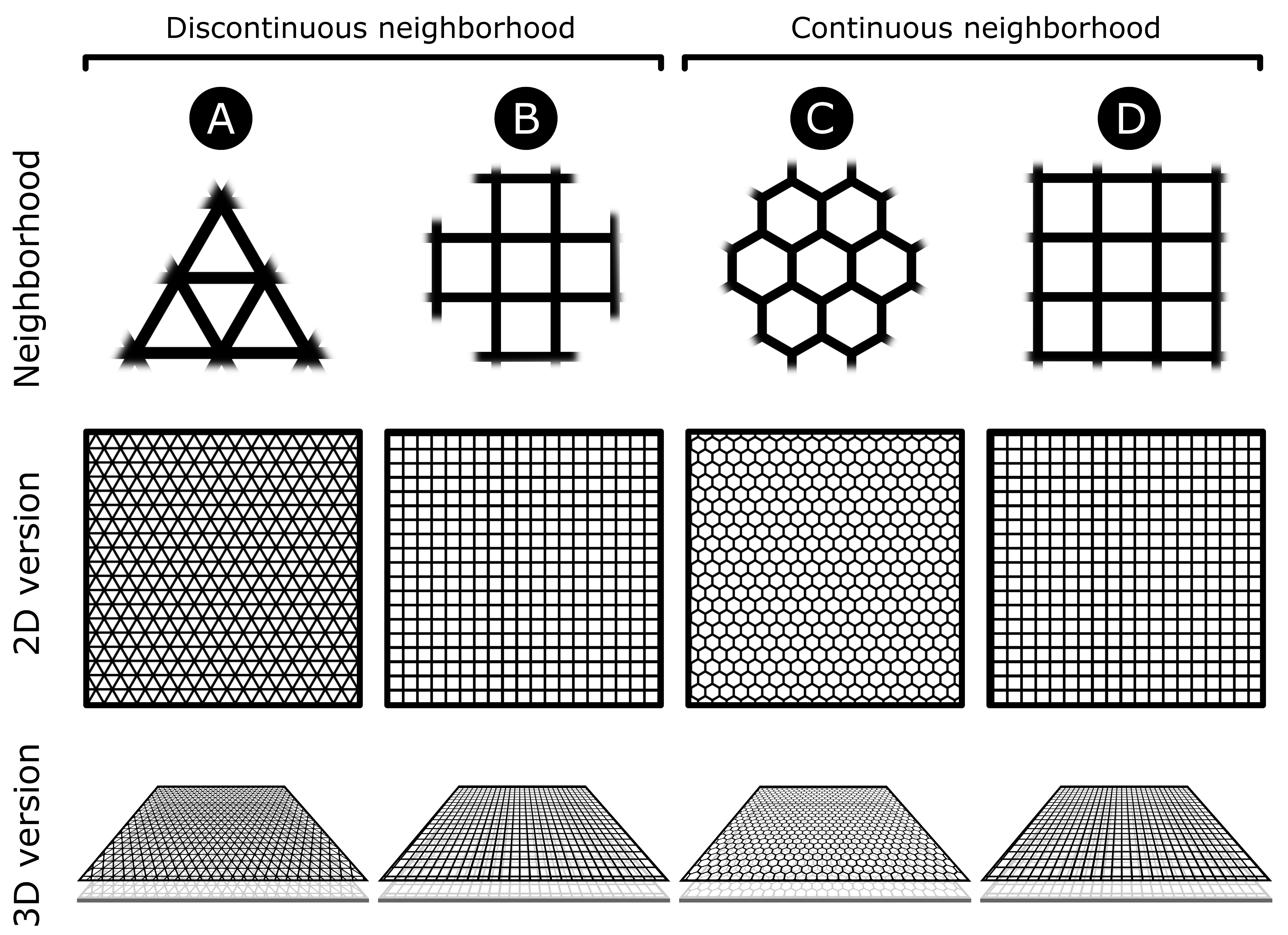

Supplement: S7 Fig — We studied four different surface geometries in which cells have three (A), four (B), six (C) and eight (D) neighbours, respectively. The surface geometries with three and four neighbours are called discontinuous, because the neighbours of a cell are not each other neighbours. The surface geometries with six and eight neighbours are called continuous, because the neighbours of a cell are each other neighbours. The surface geometries were used in a two (2D version) and three dimensional (3D version) setup. In the three dimensional setup, two cell layers are placed on top of each other (for details on the interaction structure in the 3D version see S2 Text). (TIF) [file pcbi.1004764.s007.tif]

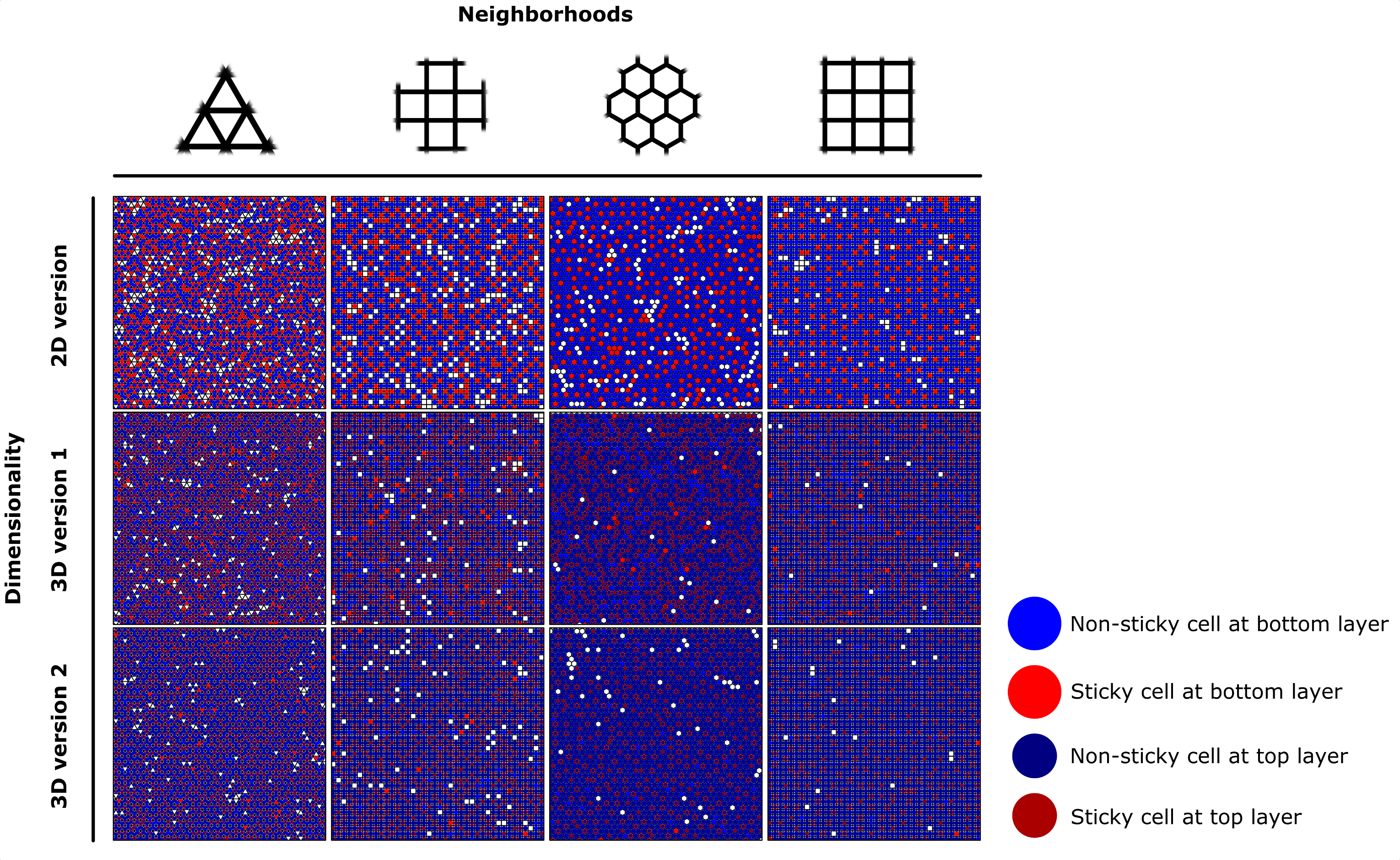

Supplement: S8 Fig — Snapshots of surface for the different surface geometries in both the two and three dimensional model implementation. For the three dimensional implementation, cell on the top layer are shown in darker colours and are slightly smaller, so that both cell layers remain visible. For a three dimensional snapshot see S9 Fig. Surface is shown at the end of evolution T = 400.000, for R = 0.5 and Pm = 0.3. (TIF) [file pcbi.1004764.s008.tif]

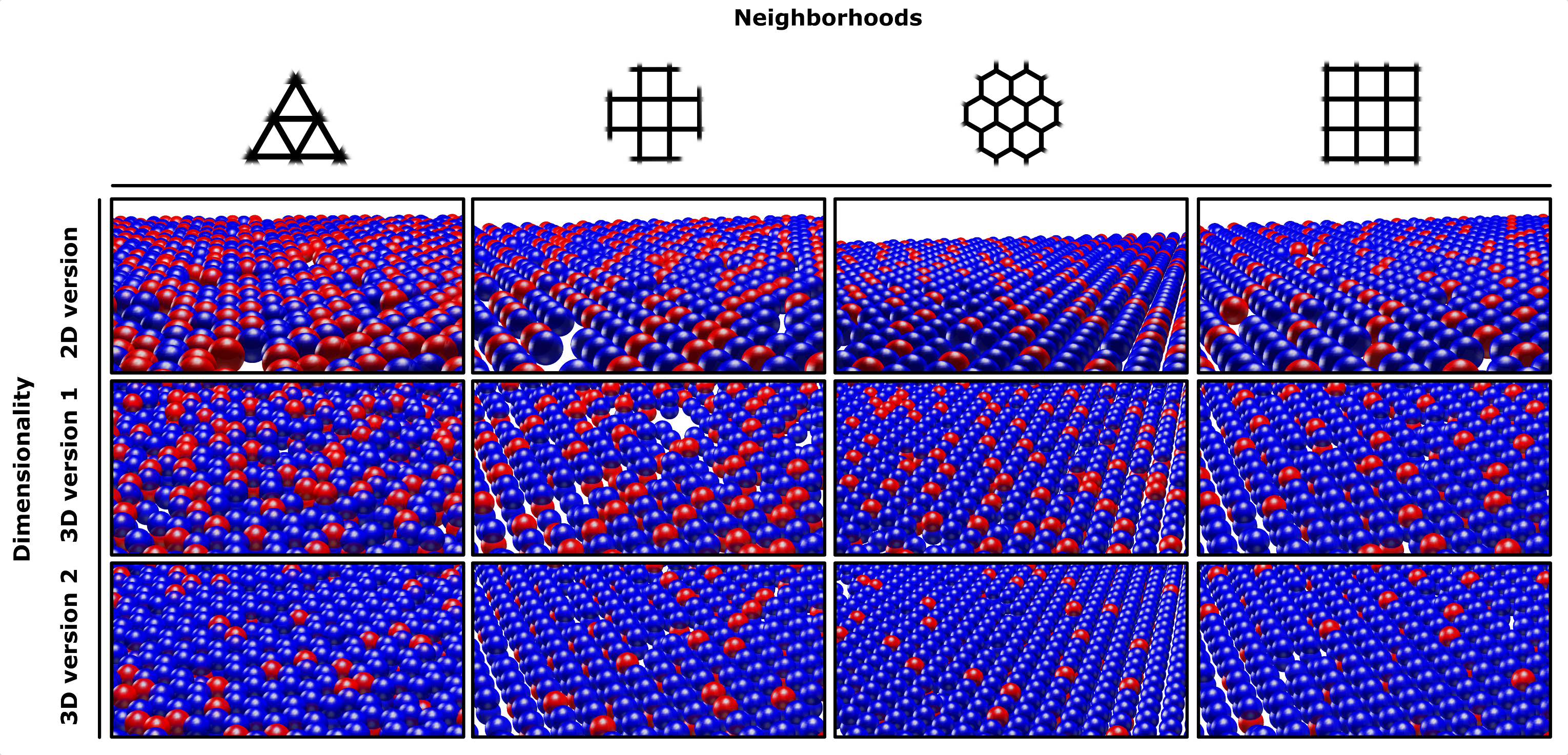

Supplement: S9 Fig — Three dimensional impression of the surface for the different surface geometries. Surface is shown at the end of evolution T = 400.000, for R = 0.5 and Pm = 0.3. (TIF) [file pcbi.1004764.s009.tif]

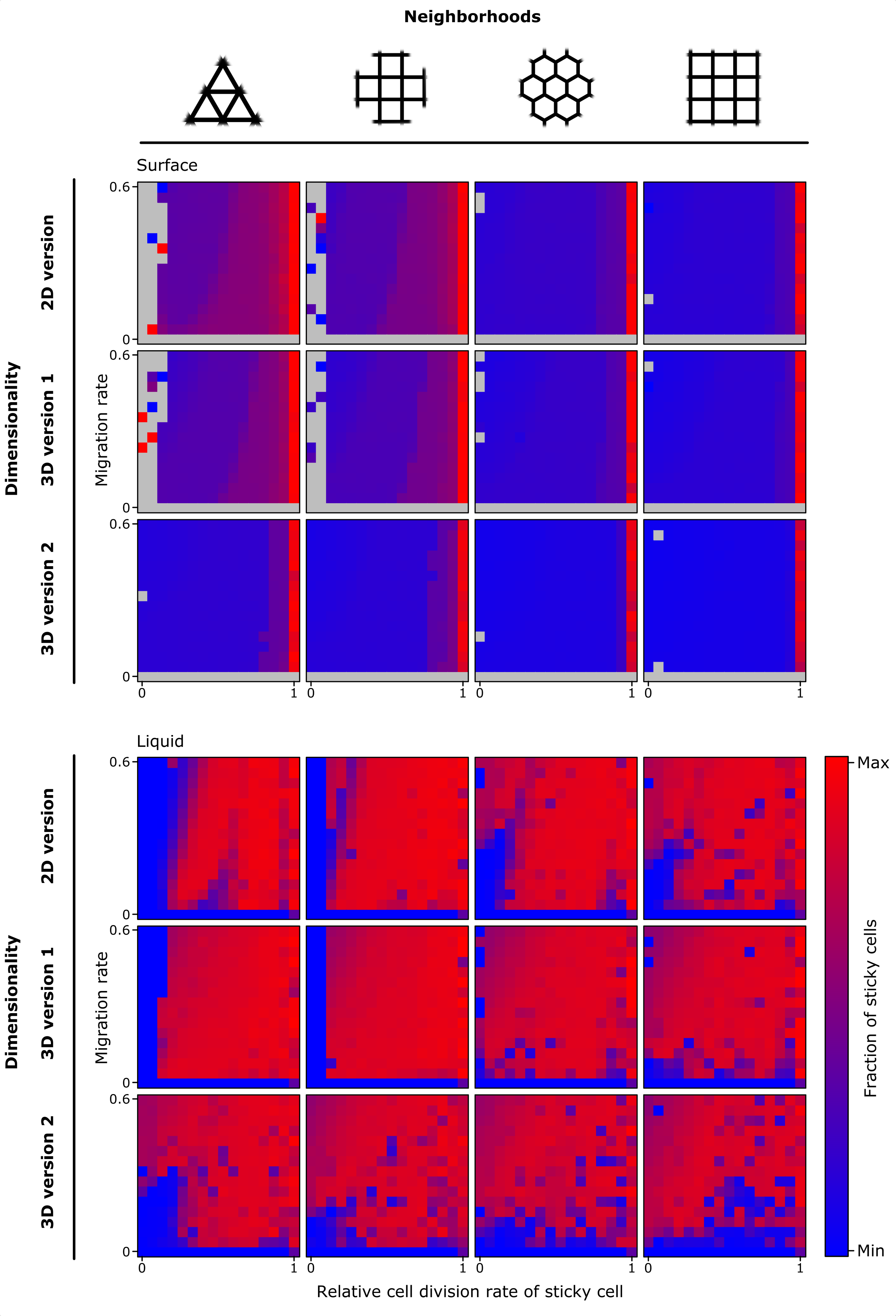

Supplement: S10 Fig — Fraction of sticky cells on surface and liquid for different parameter combinations of relative cell division rate (R) and migration rate (Pm). The fraction of sticky cells varies from no sticky cells (blue) to only sticky cells (red). When there are no cells on the surface a grey square is shown. Except for the surface geometry, all parameter settings are the same as in Fig 6. (TIF) [file pcbi.1004764.s010.tif]

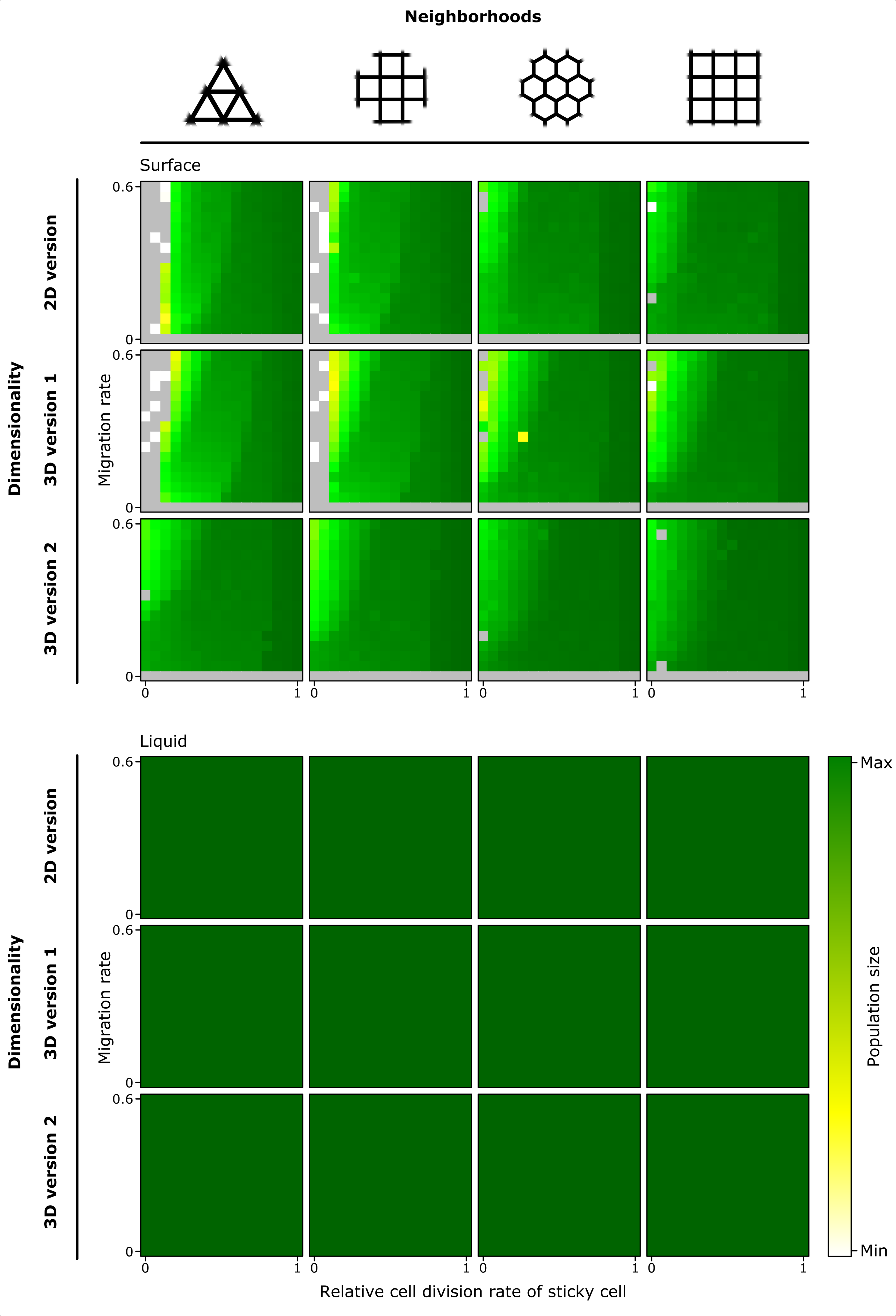

Supplement: S11 Fig — The population size on surface and liquid for different parameter combinations of relative cell division rate (R) and migration rate (Pm). The population size ranges from 0 (white) to the carrying capacity (green). When there are no cells on the surface a grey square is shown. Except for the surface geometry, all parameter settings are the same as in Fig 6. (TIF) [file pcbi.1004764.s011.tif]

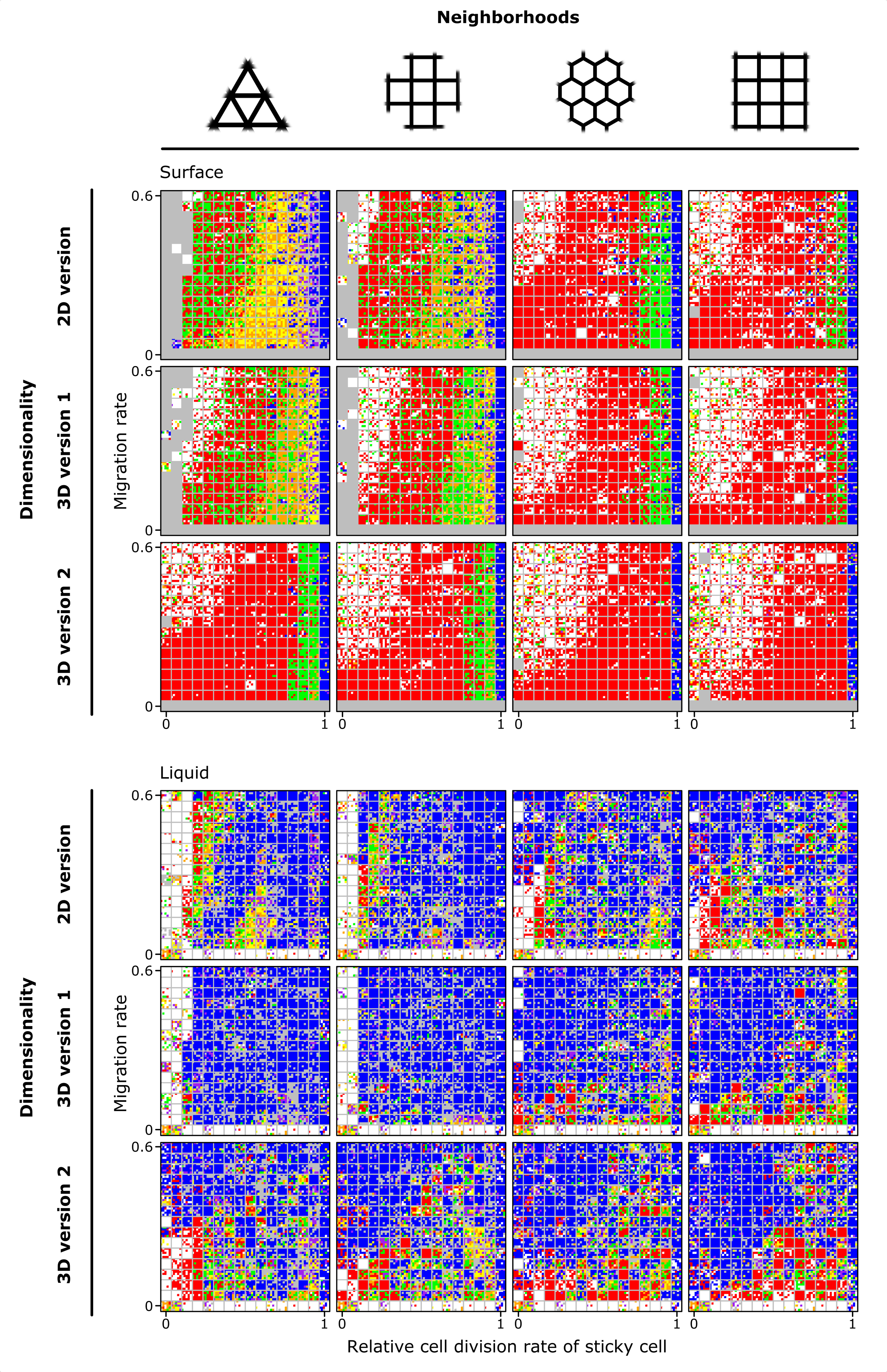

Supplement: S12 Fig — For all surface geometries and parameter combinations–Pm and R–the 25 most abundant genotypes at the end of evolution were examined. The phenotypic strategies were determined in the same way as for S5 Fig. However, in contrast to S5 Fig, cells in the different surface geometries have different number of neighbours. To facilitate comparison, we therefore determined if a genotype would differentiate on the surface when the fraction of sticky neighbours was less than n/6 (n = 1, 2, 3, 4, 5 or 6) using the same colour combination as in S5 Fig. Since we examine each genotype in both environments, every genotype is associated with two colours, one for the strategy on the surface and one for the strategy in the liquid. The strategies of the 25 most abundant genotypes are shown by the 25 colour pixels in each quadrant (i.e. every quadrant corresponds to a parameter combination). The pixels are sorted from the most abundant genotype (upper left corner of each quadrant) to the least abundant genotype (lower right corner of each quadrant). The quadrants that are entirely grey correspond to the parameter combinations in which there was no surface colonization. (TIF) [file pcbi.1004764.s012.tif]
