# Supplementary material for: Phenotypic Heterogeneity and the Evolution of Bacterial Life Cycles
Source: PLoS Comput Biol. 2016 Feb 19;12(2):e1004764. doi: 10.1371/journal.pcbi.1004764 (PMC4760940; doi:10.1371/journal.pcbi.1004764)
Supplement: S1 Table — (DOCX) [file pcbi.1004764.s013.docx]

| **Parameter** | **Default value** | **Description** |
| --- | --- | --- |
| *T* | 400000 | Number of time step simulation is ran |
| *R* | NA | Division rate of sticky cell (relative to that of non-sticky cell) |
| *P_m_* | 0.1 | Migration rate from liquid to surface |
| *P_d_* | 0.1 | Death rate |
| *K* | 5000 | Carrying capacity of cell population in liquid |
| *G* | 100 | Dimension of grid (100 x 100 = 10.000 locations) |
| *µ_r_* | 0.001 | Mutation rate |
| *µ_s_* | 0.1 | Mutation size (standard deviation of normal distribution) |
