# Supplementary material for: Phenotypic Heterogeneity and the Evolution of Bacterial Life Cycles
Source: PLoS Comput Biol. 2016 Feb 19;12(2):e1004764. doi: 10.1371/journal.pcbi.1004764 (PMC4760940; doi:10.1371/journal.pcbi.1004764)
Supplement: S1 Text — (DOCX) [file pcbi.1004764.s014.docx]

**Text S1. Illustration of surface colonization and phenotypic heterogeneity.**

To illustrate how adhesive cells can colonize a surface, even in the presence of phenotypic heterogeneity, we performed a small experiment. To this end, we used *Bacillus subtilis*, which has been intensely studied with respect to colony formation and cell differentiation [14,44] (Vlamakis *et al.* 2013; Cairns *et al.* 2014). We grew a matrix-producing wild type strain and a matrix-deficient mutant in liquid medium to investigate surface colonization at the air-liquid interface [41]. As expected from previous studies [85,86] (Bai *et al.* 1993), surface colonization only occurred in the presence of matrix-producing cells that formed floating rafts at the air-liquid interface (S1A and S1B Fig.). When using a labeled strain, in which cells express a fluorescent protein (artificially colored red in S1C Fig.), it becomes apparent that not all cells produce matrix in these floating rafts. The cells that did not produce matrix hitchhiked along with the oftentimes chaining matrix-producing cells. From this experiment it becomes clear that (i) adhesive cells are necessary for surface colonization and (ii) that not all cells that colonize the surface have to produce matrix. One should be aware that surface colonization at the air-liquid interface, like described here, acquires different biophysical properties – e.g. buoyancy – than attachment to solid surfaces.

**References**

Bai U, Mandic-Mulec I, Smith I. SinI modulates the activity of SinR, a developmental switch protein of *Bacillus subtilis*, by protein-protein interaction. Genes Dev. 1993;7: 139–148. doi:10.1101/gad.7.1.139

Cairns LS, Hobley L, Stanley-Wall NR. Biofilm formation by *Bacillus subtilis*: new insights into regulatory strategies and assembly mechanisms. Mol Microbiol. 2014;93: 587–598. doi:10.1111/mmi.12697

Vlamakis H, Chai Y, Beauregard P, Losick R, Kolter R. Sticking together: building a biofilm the *Bacillus subtilis* way. Nat Rev Microbiol. 2013;11: 157–168. doi:10.1038/nrmicro2960
