# Supplementary material for: Phenotypic Heterogeneity and the Evolution of Bacterial Life Cycles
Source: PLoS Comput Biol. 2016 Feb 19;12(2):e1004764. doi: 10.1371/journal.pcbi.1004764 (PMC4760940; doi:10.1371/journal.pcbi.1004764)
Supplement: S2 Text — (DOCX) [file pcbi.1004764.s015.docx]

**Text S2. Surface geometry and dimensionality**

The life cycles that are observed in the main text, were only examined for a hexagonal grid, using a two dimensional surface implementation. To examine if the same life cycles evolve for other grid implementations, we examine three additional surface geometries (S7 Fig.). In the two dimensional implementation (2D version) of these surface geometries, cells have respectively 3 (S7A Fig.), 4 (S7B Fig.), 6 (S7C Fig.) and 8 (S7D Fig.) neighbours. For the first two surface geometries (3 and 4 neighbours) there is a discontinuous neighbourhood, because the neighbours of a cell do not neighbour each other. For the latter two surface geometries (6 and 8 neighbours) there is a continuous neighbourhood, because the neighbours of a cell are also each other’s neighbours. In addition to the 2D version of the model, we also examined a 3D version of the model.

In the 3D version, we assume that two cell layers are placed on top of each other: the bottom and top layer. Both layers have the same surface geometry (S7 Fig). We aimed to keep the differences between the 2D and 3D version of the model as small as possible, to facilitate their comparison. Cells could only occupy the top layer, when placed on top of another cell that occurs in the bottom layer. Thus, when cells migrate from the liquid to the surface, they can either attach to the surface directly (i.e. bottom layer) or attach to another cell (i.e. top layer). If a cell in the bottom layer dies the cell on top of it sinks to the bottom layer. As a consequence, there are never vacant holes underneath cells in the top layer. Cells in the bottom layer could only migrate to the liquid or dislodge a cell to the liquid after cell division, when there is no cell placed on top of it. Thus, cells on the bottom layer face stronger constraints than cells in the top layer: when the top layer is fully occupied, cells in the bottom layer cannot divide. This could be seen in analogy to starving cells inside a densely populated colony. With respect to the other assumptions, the 3D version of the model equals the 2D version of the model.

We use two different implementations of the 3D model, in which we vary the neighbourhood of a cell. In both versions, cells in either the top or bottom layer interact with their direct neighbours that occur in the same layer (given the interaction geometry that applies, S7 Fig.). The versions differ in the interactions that occur in the z direction. In one version (3D version 1), cells can only interact with the direct neighbour that is placed above (when cell occurs in bottom layer) or below it (when cell occurs in top layer). In the other version (3D version 2), cells can interact with the entire neighbourhood that is placed above (when cell occurs in bottom layer) or above it (when cell occurs in top layer). This neighbourhood corresponds to the interaction geometry that applies to the grid, as shown in S7 Fig. Please note that, when including the full neighbourhood in the z-direction, the neighbourhood of the first two interaction geometries (S7A and S7B Fig.) becomes continuous.

Thus, in total, the following twelve implementations were tested:

1. **2D version**: *triangular lattice* with **3** neighbours.
2. **2D version**: *square lattice* with **4** neighbours.
3. **2D version**: *hexagonal lattice* with **6** neighbours.
4. **2D version**: *square lattice* with **8** neighbours.
5. **3D version 1**: *triangular lattice* with **4** neighbours (incl. one neighbour in z-direction)
6. **3D version 1**: *square lattice* with **5** neighbours (incl. one neighbour in z-direction)
7. **3D version 1**: *hexagonal lattice* with **7** neighbours (incl. one neighbour in z-direction)
8. **3D version 1**: *square lattice* with **9** neighbours (incl. one neighbour in z-direction)
9. **3D version 2**: *triangular lattice* with **7** neighbours (incl. 4 neighbours in z-direction)
10. **3D version 2**: *square lattice* with **9** neighbours (incl. 5 neighbours in z-direction)
11. **3D version 2**: *hexagonal lattice* with **13** neighbours (incl. 7 neighbours in z-direction)
12. **3D version 2**: *square lattice* with **17** neighbours (incl. 9 neighbours in z-direction)

For each model implementation, we performed 256 evolutionary simulations, one for each combination of *R* and *P_m_*. *R* was varied from 0 to 1 and *P_m_* was varied from 0 to 0.6. After 400.000 time steps the simulations were analysed in the same way as the hexagonal grid is analysed in Fig. 6 and Fig. 7 of the main text. S8 Fig. and S9 Fig. show a representative *top down* and *bird eye* view of the surface at the end of evolution (*R* = 0.5 and *P_m_* = 0.3).

**Results**

S10, S11 and S12 Fig. show the fraction of sticky cells, population size and phenotypic strategies at the end of evolution for the different model implementations. These figures correspond to Fig. 6 and 7 of the main text. In general, the outcome of evolution was robust against the model implementation. In other words, for most model implementations (i.e. interaction geometries and surface dimensionality), the same life cycles evolved as those observed in Fig. 7. Only for the *triangular* and *square* interaction geometries with discontinuous neighbourhoods – **2D version** and **3D version 1** – different life cycles evolved (model implementation (1), (2), (5) and (6); as listed above). For these model implementations, neighbouring cells are not each other’s neighbours. As a consequence, colony fission becomes impossible. When the costs of being sticky are high (low *R* values), the sticky cell cannot divide. In that case, colonies can only reproduce through fission. However, when having discontinuous neighbourhoods, colony fission is hampered. When the sticky cell in the colony centre dies, non-sticky neighbours can differentiate, but they cannot be fully surrounded by non-sticky siblings (because the neighbourhood is discontinuous). As a consequence, the sticky cells have a high risk of being parasitized by other genotypes, which makes surface colonization disadvantageous. At low *R* values, surface colonization can therefore not evolve. In contrast, for the *triangular* and *square* interaction geometries with continuous neighbourhoods – **3D version 2** – colony fission and surface colonization can evolve at low *R* values (model implementation (9) and (10)). Interestingly, the life cycles (and associated parameter ranges) that evolved in the 3D implementation of the *triangular* grid are nearly identical to those of the **2D version** of the *hexagonal grid*. In both model implementations, the neighbourhoods have approximately the same size. The fact that, in the 3D model, cells in the bottom layer cannot divide has only a small effect on the outcome of the model.

For the other model implementations the same qualitative life cycles evolved as those shown in Fig. 7. Yet, the parameter ranges that are associated with the life cycles changes with the size of a cell’s neighbourhood. As expected, colonies with a larger neighbourhood have a higher chance to be parasitized. This is for example the case for the 3D version of the *hexagonal* grid. When there is larger neighbourhood, the parameter range that is associated with *life cycle 4* increases (i.e. type of surface colonization that evolves with high parasite pressure) and the parameter range that is associated with *life cycle 3* decreases (i.e. type of surface colonization that evolves with low parasite pressure).

Thus, in conclusion, the dimensionality and geometry (given that the neighbourhood is continuous) of the surface have a fairly small effect on the outcome of evolution, but the continuity and absolute size of a cell’s neighbourhood do influence the life cycles that evolve.
